# Reactive Oxygen Species Across Death Pathways: Gatekeepers of Apoptosis, Ferroptosis, Pyroptosis, Paraptosis, and Beyond

**DOI:** 10.3390/ijms262010240

**Published:** 2025-10-21

**Authors:** Noah Sendtner, Rebecca Seitz, Noah Brandl, Martina Müller, Karsten Gülow

**Affiliations:** Department of Internal Medicine I, Gastroenterology, Hepatology, Endocrinology, Rheumatology, Immunology, and Infectious Diseases, University Hospital Regensburg, 93053 Regensburg, Germany; noah.sendtner@stud.uni-regensburg.de (N.S.); rebecca.seitz@klinik.uni-regensburg.de (R.S.); noah.brandl@stud.uni-regensburg.de (N.B.); martina.mueller-schilling@ukr.de (M.M.)

**Keywords:** regulated cell death (RCD), reactive oxygen species (ROS), apoptosis, necroptosis, ferroptosis, pyroptosis, paraptosis, autophagy

## Abstract

Reactive oxygen species (ROS) are versatile determinants of cell fate, tipping the balance between survival and death. By exceeding critical thresholds or perturbing compartment-specific signaling, ROS can initiate, modulate, or suppress regulated cell death (RCD). Importantly, their influence extends across the full spectrum of currently characterized RCD modalities. 19 distinct forms of cell death—including both long-established and recently described entities—are shaped by ROS, either as triggers, modulators, or inhibitors. Beyond pathway-specific effects, ROS promote crosstalk between death programs, enabling switches from one mode to another and determining whether outcomes are inflammatory or non-inflammatory. By systematically integrating 19 RCD types, the unifying role of ROS emerges as both gatekeeper and connector of diverse death pathways. Such a comprehensive perspective underscores the centrality of redox imbalance in cell fate control and highlights its broader implications for inflammation and disease.

## 1. Introduction

In the context of aerobic metabolism, molecular oxygen serves as the terminal electron acceptor in the mitochondrial electron transport chain, enabling efficient ATP production through oxidative phosphorylation. However, this process is not entirely efficient, and a small proportion of electrons can prematurely escape from the electron transport chain (ETC). These leaked electrons can react with molecular oxygen to form superoxide anions (O_2_•^−^), a primary type of reactive oxygen species (ROS) [1,2,3]. Importantly, ROS are not merely byproducts of mitochondrial respiration. The electron transport chain under specific physiological or stress-induced conditions requiring redox signaling can also deliberately generate them via complexes I and III of the ETC. In addition, ROS are produced through various enzymatic reactions, most notably by the NADPH oxidase (NOX) family of enzymes. In both mitochondrial and non-mitochondrial contexts, ROS serve not only as signaling molecules that regulate processes such as cell proliferation, differentiation, and death, but also as effectors of innate immune defense, particularly in phagocytic cells where they contribute to pathogen clearance via the oxidative burst [1,3,4].

Regulated cell death (RCD) is a fundamental biological process that is essential for the maintenance of tissue homeostasis and the overall survival of multicellular organisms [5]. By eliminating damaged, aged, or potentially malignant cells in a controlled manner, cell death contributes to the preservation of tissue integrity and prevents the accumulation of dysfunctional or genetically unstable cells. This tightly controlled process includes both developmentally programmed cell death—as seen during embryogenesis—and stress-induced forms triggered by environmental or intracellular signals. While programmed cell death (PCD) traditionally refers to genetically encoded cell elimination during development, the broader concept of RCD encompasses all molecularly controlled death pathways, including those activated in response to cellular damage or transformation. Together, these mechanisms ensure balanced cellular turnover and allow immune surveillance systems to eliminate cells with oncogenic mutations or irreparable damage [6].

When cellular ROS levels exceed a critical threshold, they can trigger the activation of various forms of PCD/RCD. While classical apoptosis, an orchestrated, caspase-dependent process, remains the most extensively studied form, recent years have seen the identification of numerous alternative RCD modalities, each defined by distinct molecular mechanisms [7,8]. Among these are ferroptosis, an iron-dependent form of cell death characterized by lipid peroxidation [9,10,11], and pyroptosis, a lytic and pro-inflammatory form of cell death mediated by gasdermin-induced membrane pore formation [12,13,14,15,16,17]. In addition, necroptosis, a regulated necrotic process involving receptor-interacting protein kinase 3 (RIPK3) and mixed lineage kinase domain-like protein (MLKL) [18], and parthanatos, which is driven by poly(ADP-ribose) polymerase 1 (PARP1) [19,20].

Other ROS-associated death pathways include paraptosis, marked by cytoplasmic vacuolization and mitochondrial swelling [6], lysosome-dependent cell death, where ROS promote lysosomal membrane permeabilization [21], and oxeiptosis, a recently described, non-inflammatory form of cell death triggered by high ROS concentrations and mediated via Kelch-like ECH-associated protein 1 (KEAP1) and phosphoglycerate mutase family member 5 (PGAM5)-dependent signaling [22,23]. In addition, an expanding repertoire of regulated cell death modalities such as anoikis [24], entosis [25,26], mitotic catastrophe [27], methuosis [28], cuproptosis [29], alkaliptosis [28], disulfidptosis [28], and others has been linked to redox imbalance. This diversity illustrates that ROS do not merely act within a few selected pathways but represent a common thread across almost the entire spectrum of RCD, influencing both their initiation and their inflammatory or non-inflammatory outcomes. A deeper understanding of ROS-mediated signaling pathways and their impact on distinct death programs is essential for the development of targeted therapies for diseases in which oxidative stress plays a pathogenic role, such as cancer, neurodegeneration, and chronic inflammation.

## 2. An Evolutionary Perspective on ROS as Master Regulators of Cell Fate

The pervasive role of ROS in virtually all RCD modalities can be understood within an evolutionary framework. Prior to the emergence of photosynthetic archaebacteria approximately 2.7 billion years ago, the Earth’s atmosphere was predominantly anoxic. Life at that time was strictly anaerobic, and the advent of oxygen (O_2_) produced by oxygenic photosynthesis represented a profound evolutionary challenge [1,30,31,32,33]. The absence of protective mechanisms against oxidative stress rendered oxygen highly toxic, leading to widespread extinctions. Surviving organisms adapted by developing antioxidant defenses and, eventually, by harnessing oxygen for energy production through oxidative phosphorylation [1,34]. This innovation enabled a dramatic increase in bioenergetic efficiency, but at the cost of inevitable by-products—like ROS. Over time, organisms evolved not only to defend against ROS but also to exploit them as integral components of signaling networks. ROS thus transitioned from being unwanted, potentially lethal by-products of metabolism to deliberately generated molecules that serve critical physiological functions [1,35,36,37]. This duality, damage versus signaling, provides a unique evolutionary rationale for why ROS have become central regulators of cell fate. Their chemical reactivity allows them to modify nucleic acids, lipids, and proteins, thereby influencing core cellular processes such as mitochondrial respiration, DNA repair, protein folding, and membrane integrity. At the same time, their infusibility and rapid turnover make them highly adaptable messengers capable of integrating diverse stress inputs into coherent biological outcomes. From this perspective, it is not surprising that ROS are embedded as common regulatory nodes across distinct RCD pathways. In each case, ROS function as metabolic sentinels that reflect the energetic and redox status of the cell, tipping the balance between survival and demise. The evolutionary legacy of ROS thus explains their ubiquity as fate determinants: as unavoidable products of oxygen-based life, they became both threat and opportunity, co-opted into regulatory circuits that now orchestrate cell death across species and contexts [38,39,40].

A key aspect of the evolutionary rationale for ROS as central cell fate regulators is their duality as both damaging agents and signaling molecules. On the one hand, highly reactive species such as superoxide anions (O_2_•^−^) and hydroxyl radicals (•OH) exhibit extremely short half-lives and indiscriminate reactivity with macromolecules, thereby exerting primarily cytotoxic effects. On the other hand, the relatively more stable hydrogen peroxide (H_2_O_2_) has emerged as a central redox signal with a longer half-life and the capacity to diffuse across membranes. This “Janus-faced” nature allows ROS to serve as both stress indicators and active messengers that initiate adaptive responses or trigger cell death when homeostatic thresholds are exceeded. In this way, ROS function as molecular translators of cellular stress intensity, bridging the continuum between adaptation and demise [4,35,41,42,43]. The specific generation and biochemical properties of distinct ROS , will be addressed in detail in Section 3 (Reactive Oxygen Species: Types and Cellular Sources).

Beyond their dual role as damaging and signaling molecules, ROS act as integrative nodes that converge multiple stress signals into cell fate decisions. Their ability to oxidize proteins, lipids, and nucleic acids allows ROS to modulate virtually all core cellular processes, including enzyme activity, membrane integrity, transcriptional regulation, and genome stability [44,45]. In this way, ROS serve as molecular bridges that couple environmental and intracellular cues, such as metabolic stress, infection, hypoxia, or immune activation, to the activation of RCD pathways [37,46]. This nodal function explains why diverse RCD modalities, despite their distinct molecular machineries, share ROS as common regulators. Ultimately, ROS translate heterogeneous stress inputs into a binary decision of survival versus death, thereby ensuring that cellular homeostasis and organismal integrity are maintained under fluctuating conditions.

From an evolutionary perspective, the ubiquity of ROS in RCD becomes less surprising. The bioenergetic use of oxygen inevitably leads to ROS generation as by-products of oxidative phosphorylation, compelling all aerobic organisms to evolve mechanisms to tolerate and exploit them [34]. Moreover, the physicochemical features of ROS, their versatile reactivity, render them uniquely suited to serve as universal amplifiers of stress signals across subcellular compartments [37,45]. Finally, because ROS production is tightly coupled to redox balance, they provide a direct and dynamic reflection of the metabolic state of the cell, thereby linking energy metabolism to survival decisions [35,36]. Taken together, these evolutionary constraints and opportunities have embedded ROS as universal regulators of cell fate, shaping their central role across diverse forms of RCD.

## 3. Reactive Oxygen Species: Types and Cellular Sources

A comprehensive understanding of the cellular Redox equilibrium requires knowledge of which ROS species are present in cellular systems, their physicochemical properties, and their sites of generation. In this section, we focus on the major ROS and their biological relevance, while not addressing reactive nitrogen species (RNS) to keep the scope centered on ROS and avoid unnecessary complexity.

### 3.1. Types of Reactive Oxygen Species

The predominant ROS in cells are O_2_•^−^, H_2_O_2_, and •OH. O_2_•^−^ is generated first by the monovalent reduction of molecular O_2_ and is subsequently dismutated, either spontaneously or catalyzed by superoxide dismutases (SODs), into H_2_O_2_ [1,2,47]. In cells, O_2_•^−^ and H_2_O_2_ can together produce highly reactive •OH through the Haber–Weiss reaction. This net process occurs mainly via two consecutive steps: (i) reduction of ferric to ferrous iron by O_2_•^−^ (O_2_•^−^ + Fe^3+^ → O_2_ + Fe^2+^) and (ii) the Fenton reaction, in which Fe^2+^ reacts with H_2_O_2_ to generate •OH and OH^−^ (Fe^2+^ + H_2_O_2_ → Fe^3+^ + •OH + OH^−^). The sum of these reactions yields the overall Haber–Weiss equation (O_2_•^−^ + H_2_O_2_ → O_2_ + •OH + OH^−^) [47]. Beyond O_2_•^−^, H_2_O_2_, and •OH, oxygen-derived free radicals also include peroxyl radicals (ROO•). The simplest member of this family is the hydroperoxyl radical (HOO•), the conjugate acid of O_2_•^−^ (pKa ≈ 4.8) [48]. At physiological pH, O_2_•^−^ predominates, but in acidic microenvironments or within hydrophobic phases HOO• becomes more relevant and, due to its greater membrane solubility, can participate in lipid oxidation. In cells, HOO• can be produced via the Fenton-like reduction of Fe^3+^ by H_2_O_2_ (Fe^3+^ + H_2_O_2_ → Fe^2+^ + HOO• + H^+^) [48]. Peroxyl radicals (ROO•), including the hydroperoxyl radical (HOO•), are important chain-propagating species in lipid peroxidation. This process is typically initiated when reactive species such as •OH abstract a hydrogen atom from polyunsaturated fatty acids (PUFAs), generating lipid radicals that rapidly react with O_2_ to form ROO•. These radicals convert PUFAs into lipid hydroperoxides (LOOH), which are unstable and, in the presence of transition metals, decompose into intermediates that are more reactive. Secondary products such as 4-hydroxy-2-nonenal (4-HNE) and malondialdehyde (MDA) can form adducts with proteins and nucleic acids, contributing to structural and functional damage. ROO•-driven lipid peroxidation, especially when amplified by iron-dependent redox cycling, plays a key role in cell death pathways [49]. 

### 3.2. Key Features of O_2_•^−^, H_2_O_2_, and •OH

O_2_•^−^ high reactivity results from its energetically unstable electronic configuration, and it has a short half-life of approximately 1 µs under physiological conditions. Due to its negative charge, O_2_•^−^ cannot freely traverse lipid bilayers, confining its reactivity to the immediate site of formation. Consequently, its biological impact is highly localized and is typically associated with oxidative damage to nearby proteins, lipids, or metal centers rather than with long-range signaling [1,4,50].

H_2_O_2_, although not a radical, is classified as an ROS because it participates in oxidative processes and serves as a key intermediate in ROS conversion cascades. It is significantly more stable than O_2_•^−^, with a half-life of about 1 ms, and its uncharged nature allows free diffusion across membranes via aquaporins. H_2_O_2_ preferentially oxidizes protein thiol groups, forming sulfenic acids in reactions that are generally reversible, thereby enabling regulation of redox-sensitive signaling pathways. This capacity to act in a controlled, reversible manner across cellular compartments underlies its role as a second messenger in redox biology [1,4,36,50,51].

•OH is the most reactive ROS, with a half-life of less than 1 µs, and is generated primarily via metal-catalyzed decomposition of H_2_O_2_ (e.g., Fenton chemistry). Due to its extreme reactivity and lack of enzymatic detoxification pathways, •OH reacts at diffusion-limited rates with virtually all classes of biomolecules, causing non-selective and often irreversible damage. This includes lipid peroxidation, protein carbonylation, and nucleic acid oxidation, processes that can compromise cell integrity and trigger cell death pathways [1,4,50,52,53].

### 3.3. Primary Sources of ROS

Cells harbor multiple enzymatic and non-enzymatic sources of ROS, distributed across distinct subcellular compartments and active under various physiological and pathological conditions. In this review, we focus on the major contributors—mitochondrial ETC, NOX, and cytochrome P450.

**Figure 1:**
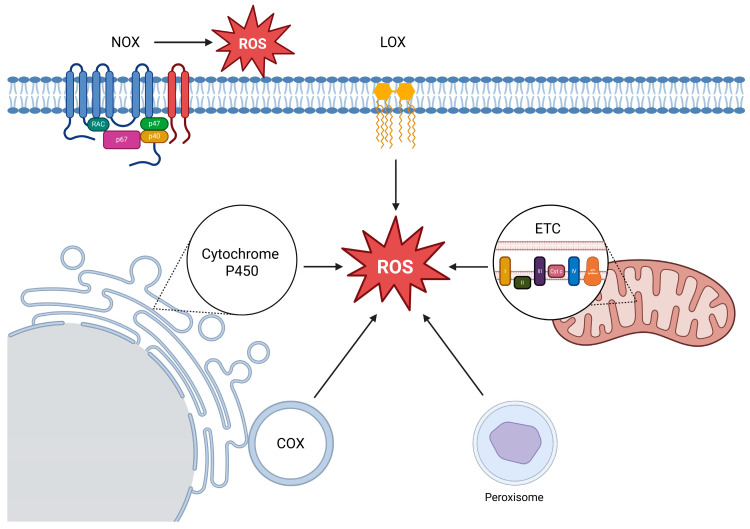
Primary sources of ROS. The three major sources of ROS are the mitochondrial electron transport chain (ETC), NADPH oxidases (NOX), and cytochrome P450 enzymes. Mitochondria generate ROS as by-products through electron leakage from the ETC. NOX enzymes transfer electrons from NADPH to O_2_, producing O_2_•^−^, which can be further converted to H_2_O_2_. Cytochrome P450 enzymes generate ROS via electron leakage during their catalytic cycle, leading to the formation of O_2_•^−^ and H_2_O_2_. In addition, lipoxygenases (LOX) and cyclooxygenases (COX) contribute to ROS production through arachidonic acid metabolism. ATP = adenosine triphosphate; COX = cyclooxygenase; Cyt c = cytochrome c; ETC = electron transport chain; LOX = lipoxygenase; NOX = NADPH oxidase; RAC = RAC GTPase; ROS = reactive oxygen species. This figure was created with BioRender (https://biorender.com); original files available on request.

Mitochondria produce ROS both as by-products of electron leakage and, under certain conditions, as deliberate signaling intermediates [1,54,55,56,57] (Figure 1). The mitochondrial ETC, located in the inner membrane, comprises five main complexes (I–V), with Complex I (NADH:ubiquinone reductase) and Complex III (ubiquinol:cytochrome c reductase) being the predominant sites of ROS formation [46,56,58,59,60]. Electron leakage at these complexes generates O_2_•^−^, which is rapidly converted to H_2_O_2_ by SOD2 in the matrix or SOD1 in the intermembrane space [61,62]. ROS production is modulated by factors such as membrane potential, redox state, and substrate availability and excessive mitochondrial ROS can cause oxidative damage to mitochondrial DNA, lipids, and proteins, contributing to mitochondrial dysfunction, cell damage, and cell death [63,64].

NOX constitute a family of membrane-associated enzymes dedicated to ROS generation (Figure 1). By transferring electrons from NADPH to O_2_, they produce O_2_•^−^, which is readily converted to H_2_O_2_ [65,66]. Multiple isoforms (NOX1–NOX5) operate in distinct cellular contexts, supporting functions ranging from microbial killing to redox signaling. DUOX1 and DUOX2, structurally related members with an additional peroxidase-like domain, primarily generate H_2_O_2_ and play roles in epithelial host defense [67]. While tightly regulated under physiological conditions, excessive NOX activity is linked to oxidative stress and cell death in diverse pathologies [66,68].

Cytochrome P450 enzymes represent an important cellular source of ROS, as “electron leakage” during their catalytic cycle leads to the formation of O_2_•^−^ and H_2_O_2_ (Figure 1). In particular, Cytochrome 2E1 exhibits a high propensity for ROS generation and thereby contributes substantially to oxidative stress [69,70,71].

Arachidonic acid (AA) metabolism via lipoxygenases (LOX) and cyclooxygenases (COX) is a relevant source of ROS in diverse tissues. Following its liberation from membrane glycerophospholipids by cytosolic phospholipase A_2_ (cPLA_2_), AA is subsequently metabolized by LOX and COX to generate bioactive eicosanoids, including prostaglandins, thromboxanes, and leukotrienes. The oxidative conversion of AA through these enzymes inherently generates ROS as secondary products [72,73]. COX enzymes are predominantly associated with the endoplasmic reticulum and nuclear envelope, whereas LOX isoforms are mainly cytosolic but translocate to membranes upon activation, providing compartmentalized regulation of eicosanoid and ROS formation [74,75].

### 3.4. Cellular Mechanisms Maintaining the Redox Homeostasis

The antioxidant defense system is compartmentalized to maintain and tightly control the redox equilibrium in distinct cellular compartments. SOD1 (Cu/Zn-SOD) is located in the cytosol and the mitochondrial intermembrane space, SOD2 (Mn-SOD) in the mitochondrial matrix, and SOD3 (EC-SOD) in the extracellular space. These enzymes catalyze the conversion of O_2_•^−^ into H_2_O_2_ [76,77,78], which is subsequently degraded by catalase in peroxisomes into H_2_O and O_2_ [79]. In parallel, the glutathione (GSH) system [80] and the thioredoxin (Trx) system provide reducing equivalents across cellular compartments: Trx1 is localized in the cytosol and nucleus, whereas Trx2 resides in mitochondria [2,81]. Together, these systems preserve redox homeostasis and control ROS levels, thereby enabling redox signaling under physiological conditions while protecting cells from oxidative damage and cell death.

Beyond their general role in maintaining redox equilibrium, antioxidant enzymes also exert modality-specific effects on RCD. SODs convert O_2_•^−^ into H_2_O_2_, thereby not only limiting local oxidative damage but also shaping H_2_O_2_ availability as a second messenger that can modulate apoptotic signaling [35,44]. Catalase, by degrading H_2_O_2_ in peroxisomes, prevents its accumulation to cytotoxic levels and thereby counteracts apoptotic and necroptotic initiation [82]. In contrast, the GSH and Trx systems are particularly relevant for ferroptosis, where GSH-dependent GPX4 detoxifies lipid peroxides, and Trx reduces oxidized proteins, collectively suppressing lipid peroxidation-driven death [83,84]. Under conditions of GSH depletion or Trx inhibition, ferroptosis is strongly amplified, whereas preserved activity of these systems raises the threshold for lipid peroxide–induced damage. Thus, by controlling the quality and quantity of ROS, these antioxidant defenses not only maintain homeostasis but also fine-tune the balance between survival and the activation of distinct RCD programs. Similar principles extend to other RCD modalities: redox buffering can delay mitochondrial permeability transition-driven necrosis [85,86], modulate lysosomal destabilization [21,87], or influence immune-related forms of cell death such as pyroptosis [88,89] and NETosis [90,91]. Thus, while the molecular details differ, antioxidant defenses consistently fine-tune the thresholds at which ROS shift from physiological signals to lethal triggers across diverse forms of regulated cell death.

The transition from physiological ROS signaling to oxidative stress and irreversible cell damage is not defined by a fixed concentration threshold but represents a dynamic continuum shaped by the antioxidant defense capacity of each cell type and tissue [34,35,36]. Basal antioxidant systems, such as SOD, catalase, GSH, and Trx, differ substantially between cell lineages and even adapt to environmental conditions [45,92]. Stress exposure itself induces transcriptional upregulation of protective proteins, thereby shifting the tolerance window for ROS and delaying the onset of oxidative damage [93]. Consequently, whether ROS act as second messengers or as cytotoxic agents depends less on absolute levels than on the balance between ROS generation and context-specific antioxidant defenses. Thus, ROS concentrations that act as signals in one context can trigger cell death in another, underscoring the role of the cellular context in ROS-dependent fate decisions [34,35,36].

## 4. ROS-Dependent Pathways of Regulated Cell Death

For decades, cell death was classified into two categories: necrosis, considered an uncontrolled and pro-inflammatory process, and apoptosis, viewed as a tightly regulated, non-inflammatory program. This view has fundamentally shifted with the recognition that cells can undergo a broad spectrum of RCD modalities, each defined by specific molecular machinery and signaling networks [5,6]. To date, at least two dozen forms of RCD have been described, and the number continues to grow as novel mechanisms and cross-regulatory interactions are uncovered [94,95]. Importantly, the immunological consequences of RCD vary greatly. Apoptosis is generally non-inflammatory [5] and often associated with immune tolerance [96,97], whereas several other modalities, including immunogenic cell death (ICD), actively stimulate immune responses by releasing danger signals and pro-inflammatory mediators [98]. Thus, the mode of RCD is a decisive factor for tissue homeostasis, inflammation, and host defense. Many of these pathways are either directly initiated or modulated by oxidative signals, highlighting ROS as central regulators of cell fate decisions.

### 4.1. ROS as Modulators of Apoptosis

**Figure 2:**
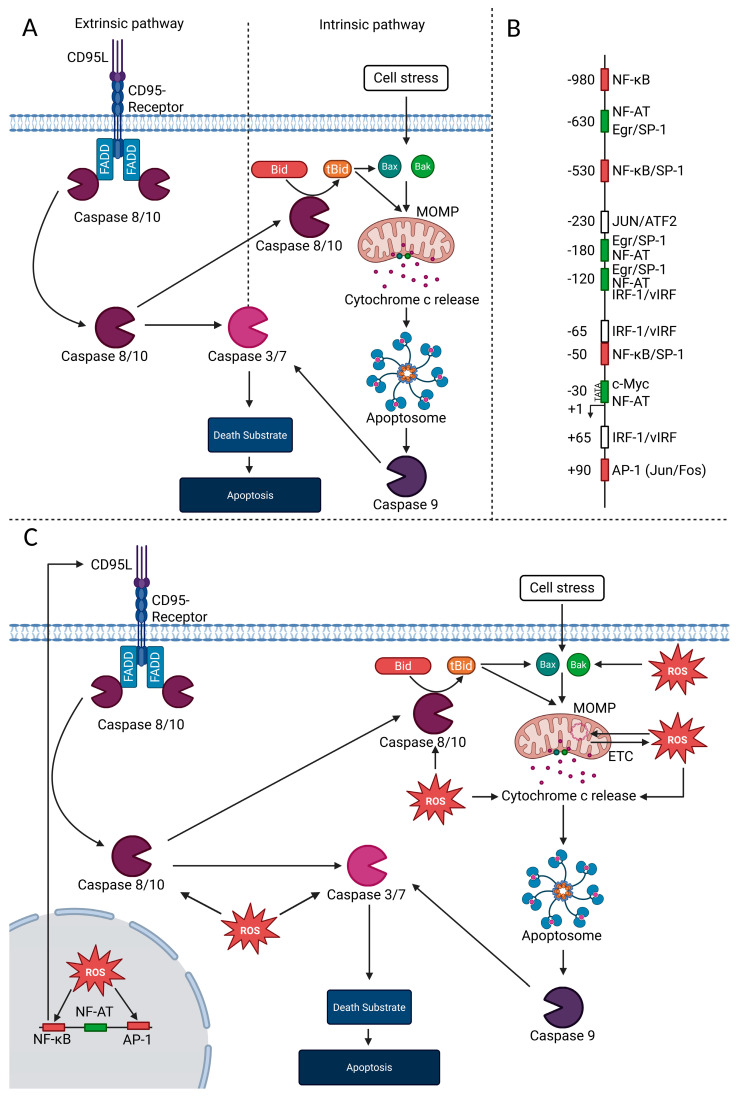
Apoptosis. (**A**) Extrinsic vs. intrinsic pathways of apoptosis. Activation of different caspases leads to degradation of various cellular structures. (**B**) Schematic illustration of CD95L Promotor. In red, binding sites of redox-sensitive transcription factors; in green, binding sites of calcium-dependent transcription factors. (**C**) Influence of ROS and cell stress on apoptosis. AP-1 (Jun/Fos) = activator protein 1; Bak = Bcl-2 homologous antagonist/killer; Bax = Bcl-2-associated X protein; Bid = BH3 interacting domain death agonist; CD95L = CD95 (Fas) ligand; Egr/SP-1= early growth response/specificity protein-1; ETC = electron transport chain; FADD = Fas-associating death domain-containing protein; IRF-1/vIRF = interferon regulatory factor 1/viral interferon regulatory factor; JUN/ATF2 = JUN/Activating transcriptions factor 2; MOMP = mitochondrial outer membrane permeabilization; NF-AT = nuclear factor of activated T cells; NF-κB = nuclear factor kappa-light-chain-enhancer of activated B cells; ROS = reactive oxygen species; tBID = truncated BH3 interacting domain death agonist. This figure was created with BioRender (https://biorender.com); original files available on request.

Apoptosis represents one of the best-characterized forms of RCD and can be initiated through two major pathways: the extrinsic (death receptor–mediated) and the intrinsic (mitochondrial) route (Figure 2A). Both pathways converge on the activation of caspases, which execute the stepwise degradation of cellular structures [5,99,100].

#### 4.1.1. ROS and the Regulation of Death Ligands in Extrinsic Apoptosis

In the extrinsic pathway, apoptosis is triggered by members of the tumor necrosis factor (TNF) family of death ligands, such as CD95 ligand (CD95L), TNF, or TRAIL, which engage their cognate receptors and initiate the formation of death-inducing signaling complexes [101,102]. Death receptors are expressed on virtually all cell types, but to avoid unintended induction of apoptosis, the expression of their ligands is tightly controlled. CD95L represents a well-studied example, as its promoter exhibits a complex architecture (Figure 2B) [5,103,104,105,106,107]. Among the transcription factors that regulate CD95L, nuclear factor of activated T cells (NFAT), activator protein 1 (AP-1), and nuclear factor kappa-light-chain-enhancer of activated B cells (NF-κB) constitute a minimal requirement for transcriptional activation (Figure 2B) [57,68,108]. NFAT is stimulated by cytosolic Ca^2+^ release, while ROS are key regulators of both AP-1 and NF-κB. AP-1 activity is strongly enhanced by ROS, in particular by H_2_O_2_ [3], which can induce the expression of c-Fos and c-Jun [109,110] as well as activate upstream MAPK cascades including ERK, JNK, and p38 [111,112,113]. A well-established mechanism involves ROS-dependent activation of apoptosis signaling kinase 1 (ASK1), which drives JNK and p38 signaling and thereby promotes c-Jun phosphorylation [114]. In addition, ROS-mediated inactivation of MAPK phosphatases further sustains AP-1 activity. Thus, ROS act at multiple levels to amplify AP-1-dependent transcription [115,116]. Similarly, moderate H_2_O_2_ level enhance NF-κB translocation into the nucleus, and inhibition of NF-κB activity by antioxidants highlights its ROS dependency [3,117,118]. ROS promote NF-κB activity by driving IκB degradation and facilitating nuclear translocation [113,119,120,121,122]. A pro-oxidative cytosolic milieu supports this process, whereas within the nucleus NF-κB must be reduced, for instance, by thioredoxin-1 (Trx-1), to bind DNA efficiently [122]. These observations emphasize the importance of the spatial distribution of ROS in shaping NF-κB-dependent transcription. Thus, ROS signaling is linked to CD95L expression, death receptor clustering, and subsequent caspase activation [62,68,108,123,124].

#### 4.1.2. ROS and Bcl-2 Family-Dependent Control of Intrinsic Apoptosis

The intrinsic apoptotic pathway is initiated at the mitochondria and tightly controlled by the B-cell lymphoma 2 (Bcl-2) family of proteins, which includes both pro-apoptotic members such as Bcl-2-associated X protein (Bax) and Bcl-2 homologous antagonist/killer (Bak) and anti-apoptotic proteins such as Bcl-2 and B-cell lymphoma-extra-large (Bcl-XL). Their balance determines mitochondrial outer membrane permeabilization (MOMP) and the subsequent release of cytochrome c. ROS have emerged as key regulators of this system. Elevated ROS levels modulate the expression and activity of Bcl-2 proteins through posttranslational modifications such as phosphorylation and ubiquitination, thereby destabilizing anti-apoptotic members and promoting the accumulation of pro-apoptotic factors [125,126,127,128]. Moreover, mitochondrial ROS production is enhanced by anti-apoptotic proteins like Bcl-2 and Bcl-XL, while pro-apoptotic Bax and Bak limit ROS generation, creating a feedback loop between redox state and apoptosis regulation [129]. Recent reviews emphasize that ROS influence Bcl-2 family function not only by affecting mitochondrial respiration but also by altering signaling pathways and chromatin regulation, underlining their central role in the initiation and amplification of intrinsic apoptosis [39,130].

A central mechanism by which ROS regulate anti-apoptotic BCL-2 family proteins is through modulation of the transcription factor NF-κB. Low to moderate levels of ROS, particularly H_2_O_2_, enhance NF-κB activation. Once activated, NF-κB induces the transcription of several anti-apoptotic genes, most prominently Bcl-xL. Bcl-xL has been identified as a bona fide NF-κB target gene, with functional κB-binding sites in its promoter required for inducible expression [131,132]. This redox-sensitive regulation highlights how NF-κB links oxidative signals to the expression of anti-apoptotic BCL-2 proteins, thereby raising the threshold for intrinsic apoptosis (Figure 2C). Pro-apoptotic BCL-2 proteins such as Bax, Bak, and BH3-only proteins (e.g., Bcl-2 Interacting Mediator of cell death (Bim), Bcl-2-modifying factor (BMF), p53 upregulated modulator of apoptosis (PUMA), phorbol-12-myristate-13-acetate-induced protein 1 (NOXA)) integrate redox cues to control MOMP. Direct effects include oxidative stress-driven conformational activation of Bax via its conserved Cys62 and high levels of ROS-promoted disulfide-dependent Bax dimerization that facilitates mitochondrial translocation [133]. Upstream signaling effects arise when oxidative stress-activated JNK phosphorylates BH3-only proteins (Bim/Bmf), releasing them from cytoskeletal anchors and engaging the Bax/Bak machinery [134]. Transcriptional effects link ROS-elicited DNA damage to the p53 network, which stabilizes and activates p53, thereby upregulating PUMA and NOXA—canonical drivers of intrinsic apoptosis. Importantly, ROS-induced DNA damage has been shown to require p53 for full transcriptional induction of these BH3-only proteins, and genetic evidence demonstrates that oxidative-stress-induced apoptosis depends on both NOXA and PUMA [135,136]. In line with these findings, work on the p53 family network has emphasized that interference with p53 and its homologs p63 and p73 critically alters cell death sensitivity, highlighting the importance of intact p53-family signaling for apoptosis induction under stress conditions [137,138]. At mitochondria, lipid oxidation creates a permissive platform: cardiolipin is peroxidized by cytochrome-c’s cardiolipin-oxygenase activity, which enables truncated Bid (tBid) docking and promotes Bax/Bak activation [139]. This mechanism provides a direct link between extrinsic and intrinsic apoptosis, as BH3 interacting domain death agonist (Bid)—cleaved by caspase-8 in death receptor signaling—is activated to tBid, thereby connecting ROS-driven mitochondrial events with death receptor pathways [99]. Together, these redox-dependent mechanisms lower the threshold for MOMP and amplify intrinsic apoptosis.

#### 4.1.3. ROS and the Regulation of Caspase Activity

In both the extrinsic and intrinsic pathways, initiator caspases are activated within large signaling platforms, the death-inducing signaling complex (DISC) for caspase-8 and -10, and the apoptosome for caspase-9. Once processed, these initiator caspases cleave and activate the effector caspases-3 and -7, which execute the apoptotic program by degrading key structural and regulatory proteins [6,99,140,141]. Beyond upstream control, ROS fine-tune caspase initiation and execution via thiol chemistry at catalytic cysteines. S-glutathionylation inhibits caspase-3 and can also block cytochrome-c/Deoxyadenosine triphosphate (dATP)-driven processing of procaspase-3 and -9; inhibition is reversible with thiol reductants [142]. Direct thiol oxidation/disulfide formation likewise prevents procaspase-3 processing [143]. S-nitrosylation suppresses multiple nodes: caspase-8 (interfering with Bid cleavage), caspase-9 (downstream of cytochrome c release), and caspase-3 (including at its active-site thiol; mass spectrometry shows S-nitrosylation and mixed disulfides with glutathione); these effects are reversible with reducing agents [144]. Among NF-κB target genes, the cellular inhibitors of apoptosis proteins (cIAPs), particularly cIAP2, serve as redox-sensitive survival checkpoints. ROS levels moderate the balance: under moderate pro-oxidative conditions, NF-κB is activated and induces cIAP2 expression, reinforcing survival by inhibiting apoptotic caspase activity [122]. cIAP2 and cIAP1, as redundant NF-κB targets, are critical for propagating NF-κB signaling by promoting Receptor-interacting serine/threonine-protein kinase (RIPK) 1 ubiquitination at TNF receptor complexes, thus preventing apoptosis initiation [145,146]. Conversely, excessive ROS can inhibit NF-κB DNA binding, thereby blocking cIAP2 induction and tilting cells toward caspase activation and apoptosis [122,147].

Overall, apoptosis therefore illustrates how ROS integrate into signaling cascades as either required, optional, or inhibitory elements depending on stimulus strength and redox balance (Table 1).

### 4.2. ROS as Modulators of Anoikis

Anoikis (literally meaning “death from homelessness”) is a specialized form of apoptosis that occurs when anchorage-dependent cells lose adhesion to the extracellular matrix (ECM). First described by Frisch and Francis (1994), it is now recognized as an apoptotic program involving intrinsic and/or extrinsic caspase pathways upon detachment [24,156,157]. Since anoikis is executed through apoptosis, the general effects of ROS on extrinsic and intrinsic death signaling (covered in the previous chapter on apoptosis) apply here as well. What distinguishes anoikis is its close dependence on integrin-mediated survival signaling. In adherent cells, integrins activate pro-survival pathways through the focal adhesion kinase (FAK) and the proto-oncogene tyrosine-protein kinase Src (Src). In addition, integrin signaling engages the Ras-related C3 botulinum toxin substrate 1 (Rac1), which stimulates NOX to produce ROS. These ROS promote Src-dependent activation of the epidermal growth factor receptor (EGFR), thereby reinforcing survival pathways and helping cells resist anoikis [158,159]. Conversely, loss of adhesion disrupts these pathways, is accompanied by mitochondrial dysfunction and elevated ROS, and promotes detachment-induced apoptosis/anoikis; antioxidants or inhibition of ROS production mitigate this response [160]. Thus, ROS occupy a dual role in anoikis, acting both as downstream signals of integrin-mediated survival pathways and as inducers of mitochondrial dysfunction once adhesion is lost, thereby critically shaping the susceptibility of cells to detachment-induced death.

### 4.3. ROS as Modulators of Necroptosis

Necroptosis is a regulated form of necrotic cell death executed by the kinases RIPK1 and RIPK3 and the downstream effector mixed lineage kinase domain like pseudokinase (MLKL) [18,161]. It is typically initiated by TNF receptor 1 (TNFR1) signaling: upon TNF binding, receptor complex I forms at the membrane, and the release or deubiquitination of RIPK1 allows the assembly of cytosolic death-inducing platforms [18,161]. Two alternative cytosolic complexes can create: the TNFR1-associated death domain protein (TRADD)–Fas-Associated Death Domain Protein (FADD)–caspase-8 complex (often referred to as the TNF-DISC, complex IIa), promoting apoptosis, or the RIPK1-based Ripoptosome (complex IIb), which functions as a molecular switch between apoptosis and necroptosis [18,162,163]. Active caspase-8 prevents necroptosis by cleaving RIPK1/RIPK3, whereas its inhibition permits RIPK1–RIPK3 interaction, formation of the necrosome, and phosphorylation-driven oligomerization of MLKL, culminating in plasma membrane disruption [18,161]. The Ripoptosome, first described by Martin Leverkus and colleagues, is a ~2-MDa complex containing RIPK1, FADD, caspase-8, and cFLIP isoforms, and its assembly is tightly regulated by cIAPs and FLICE-like inhibitory protein (cFLIP), thus critically determining the balance between apoptosis and necroptosis [122,163,164]. Accumulating evidence indicates that ROS can act as modulators of necroptotic signaling, either by amplifying RIPK1/RIPK3 activity or by contributing to downstream execution processes, thereby linking metabolic and oxidative stress to the regulation of necroptosis.

**Figure 3:**
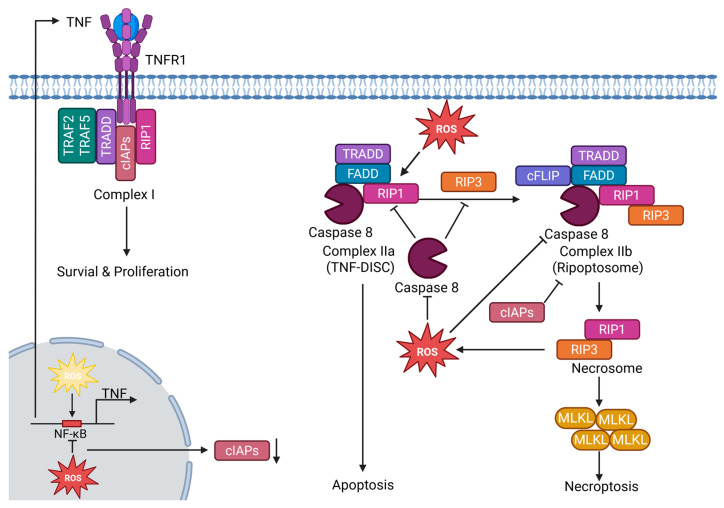
Necroptosis. Necroptosis is initiated via TNF signaling. The release or deubiquitination of RIPK1 enables the assembly of cytosolic death-inducing platforms. Two cytosolic complexes can form: the TNF-DISC (Complex IIa), in which caspase-8 is active and promotes apoptosis; and the RIPK1-based ripoptosome (Complex IIb), where caspase-8 is inactive and which functions as a molecular switch between apoptosis and necroptosis. cIAPs = cellular inhibitor of apoptosis proteins; cFLIP = FLICE-like inhibitory protein; DISC = death-inducing signaling complex; FADD = Fas-associating death domain-containing protein; MLKL = mixed lineage kinase domain like pseudokinase; NF-κB = nuclear factor kappa-light-chain-enhancer of activated B cells; RIP1/3 = receptor-interacting protein kinase 1/3; ROS = reactive oxygen species; TNF = tumor necrosis factor; TNFR1 = tumor necrosis factor receptor 1; TRADD = TNF receptor type 1-associated death domain protein; TRAF2/5 = TNF receptor-associated factor 2/5. This figure was created with BioRender (https://biorender.com); original files available on request.

Mechanistically, ROS intersects with necroptosis at multiple nodes. Upstream, mitochondrial ROS oxidize specific cysteines in RIPK1, promoting RIPK1 autophosphorylation on S161, which is essential for recruiting RIPK3 to the necrosome and establishing a positive feedback loop that amplifies necroptotic signaling [165]. Downstream, RIPK3 reprograms cellular metabolism to increase mitochondrial ROS, most prominently by directly phosphorylating the pyruvate dehydrogenase complex to boost aerobic respiration, and this step requires MLKL for efficient induction of respiration and ROS [166]. Earlier work also demonstrated that RIPK3 promotes aerobic metabolism through the activation of metabolic enzymes, thereby contributing to TNF-induced necrosis [167] (Figure 3). High ROS levels directly suppress apoptotic caspase activity through reversible thiol modifications of their catalytic cysteines (e.g., oxidation, glutathionylation). In the context of death-receptor signaling, such redox-mediated caspase-8 inhibition removes a key brake on RIPK1/RIPK3, favoring necroptosis; indeed, the physiological oxidant hypothiocyanous acid directly oxidizes and inhibits caspase-8 and thereby enables TNF-mediated necroptosis [168] consistent with the established requirement that caspase-8 activity restrains necroptosis [169,170] (Figure 3).

TNF is a principal trigger of necroptosis via TNFR1, driving RIPK1–RIPK3 necrosome formation and MLKL activation when apoptotic/survival checkpoints (e.g., caspase-8 or cIAPs) are inhibited [18,163,171,172]. TNF expression is tightly tuned by redox cues: ROS modulate NF-κB signaling, and NF-κB directly drives human TNF transcription through κB sites in the TNF promoter [117,147,173,174]. Of note, a pro-oxidant milieu in the cytosol facilitates NF-κB activation and nuclear translocation [117,122], whereas excessive ROS levels or an oxidative environment within the nucleus inhibit NF-κB DNA binding and transcriptional activity [122,175,176]. Such impairment of NF-κB-dependent transcription includes reduced expression of cIAPs, which normally suppress Ripoptosome assembly. In line with this, Trx-1 inhibition by dimethyl fumarate triggers Ripoptosome formation and induces a mixed cell death phenotype with both apoptotic and necroptotic features [122,177,178]. Since NF-κB induces anti-necroptotic factors such as c-FLIP and IAPs, this redox tuning can shift TNF outcomes toward survival or, if pro-survival signaling is blunted, toward necroptosis [179,180] (Figure 3). Thus, ROS emerge as central modulators of necroptosis, capable of shifting the balance between survival, apoptosis, and necroptosis. A comparative overview of these context-dependent roles of ROS in necroptosis is provided in Table 2.

### 4.4. ROS as Modulators of Ferroptosis

**Figure 4:**
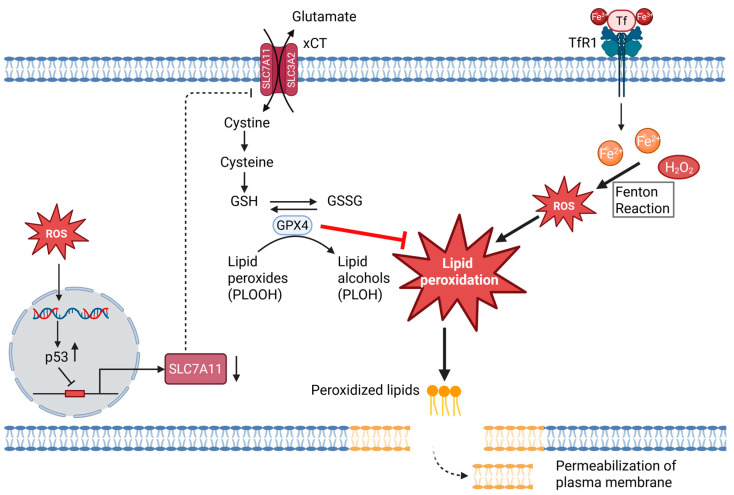
Ferroptosis. Ferroptosis is an iron-dependent regulated necrotic cell death and is triggered by inhibition of xCT antiporter or by inactivation of GPX4. Free Fe^2+^ and H_2_O_2_ initiate the Fenton reaction and generate ROS, which leads to lipid peroxidation. ROS therefore functions as a byproduct as well as an active driver of ferroptosis. Peroxidized lipids accumulate and permeabilization of the plasma membrane occurs, which drives the cell into ferroptitic cell death. Fe^2+^ = ferrous; Fe^3+^ = ferric; GPX4 = phospholipid hydroperoxide glutathione peroxidase 4; GSH = reduced glutathione; GSSG = oxidized glutathione; H_2_O_2_ = hydrogen peroxide; p53 = transcription factor p53; PLOH = lipid alcohols; PLOOH = lipid peroxides; ROS = reactive oxygen species; SLC3A2 = heavy chain of xCT antiporter; SLC7A11 = light chain of xCT antiporter; Tf = transferrin; TfR1 = transferrin receptor 1; xCT = cystine/glutamate antiporter system. This figure was created with BioRender (https://biorender.com); original files available on request.

Ferroptosis is an iron-dependent form of regulated necrotic cell death defined by the lethal accumulation of phospholipid hydroperoxides in cellular membranes [181,182]. Biochemically and genetically, it is distinct from apoptosis and necroptosis and is triggered either by inhibition of cystine import through the cystine/glutamate antiporter system xCT (a plasma membrane transporter composed of the light chain SLC7A11 and the heavy chain SLC3A2, which exchanges extracellular cystine for intracellular glutamate) or by direct inactivation of the lipid repair enzyme phospholipid hydroperoxide glutathione peroxidase 4 (GPX4) [181,183]. GPX4 uses glutathione (GSH) to detoxify lipid peroxides, and loss of GPX4 activity or depletion of GSH is sufficient to drive ferroptosis [183]. Fe^2+^ facilitates propagation of lipid peroxyl radicals and supports lipoxygenase-dependent peroxidation. In particular, a 15-lipoxygenase (15-LOX)–(phosphatidylethanolamine binding protein 1) PEBP1 complex generates pro-ferroptotic phosphatidylethanolamine hydroperoxides [182,184]. Morphologically, ferroptotic cells display shrunken mitochondria with condensed membranes and reduced or absent cristae, typically without nuclear fragmentation, distinguishing this process from other forms of regulated cell death [181] (Figure 4).

ROS are not only byproducts but also active drivers of ferroptosis. In particular, lipid ROS accumulation is the defining execution signal of this pathway, and its amplification requires both Fe^2+^-catalyzed Fenton chemistry and enzymatic lipid peroxidation. Within cells, “free” Fe^2+^ is highly reactive and therefore largely sequestered by proteins and ligands. Only a small redox-active fraction, the so-called labile iron pool (LIP), remains available, with Fe^2+^–glutathione proposed as a key LIP species [185,186,187]. H_2_O_2_ is generated by mitochondrial respiration and NOX and, at low concentrations, functions as a controlled second messenger in redox signaling [1,3,41]. Co-existence of Fe^2+^ and H_2_O_2_ can, however, initiate the Fenton reaction, producing •OH that indiscriminately attack membrane lipids and proteins, thereby promoting ferroptosis [41,188]. Cells thus tightly regulate both LIP and H_2_O_2_ to prevent uncontrolled radical chemistry, as demonstrated by the finding that NF-κB inhibition in cutaneous T-cell lymphoma leads to ROS- and iron-dependent cell death due to disturbed iron/ROS homeostasis [53,189] (Figure 4).

While the labile iron pool and H_2_O_2_ critically determine the oxidative burden that drives lipid peroxidation, the cell’s ability to counteract this threat depends on the availability of cysteine and glutathione. This protective axis is maintained by the cystine/glutamate antiporter system xCT, which fuels GSH synthesis and thereby enables GPX4 to detoxify lipid hydroperoxides [190,191] (Figure 4). Thus, system xCT activity indirectly sustains GPX4 function and protects against ferroptosis [181,183]. At the transcriptional level, oncogenic and stress pathways increase the expression of SLC7A11, a subunit of the transporter, to enhance resistance to ferroptosis. Conversely, p53 suppresses SLC7A11 expression, thereby promoting ferroptosis [192,193].

Given that cystine/glutamate antiporter system xCT, supplies cysteine for GSH synthesis, the key determinant of whether lipid peroxides remain controlled or become lethal is GPX4 (Figure 4). GPX4 is a selenoenzyme that directly reduces phospholipid hydroperoxides (PLOOH) in membranes to the corresponding alcohols (PLOH), using GSH as the electron donor [84,194]. Genetic or pharmacologic inactivation of GPX4 causes the accumulation of lipid peroxides and triggers ferroptosis, establishing GPX4 as a central negative regulator of this death pathway [181,183]. In vivo, inducible ablation of GPX4 leads to acute renal failure with characteristics consistent with ferroptotic damage, highlighting the non-redundant role of GPX4 in tissues [195]. The essential nature of GPX4 for mammalian viability is further highlighted by embryonic lethality upon GPX4 loss in mice [196]. Mechanistically, ferroptosis can be induced either upstream by depleting GSH or downstream by directly impairing of the peroxidase activity of GPX4, both converging on unchecked lipid peroxidation as the execution signal [181]. An overview of the regulatory levels at which ROS act as required, optional, or shifting factors in ferroptosis is summarized in Table 3.

### 4.5. ROS as Modulators of Pyroptosis

**Figure 5:**
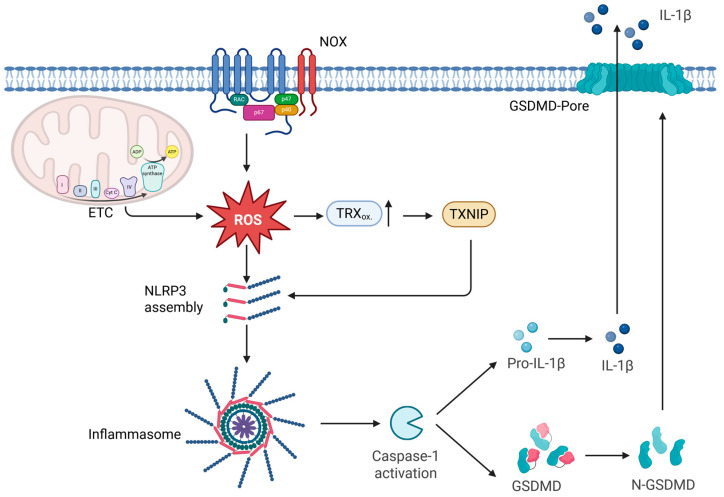
Pyroptosis. Mitochondrial ROS from electron leakage of the ETC and ROS generated from NOX promote NLRP3 inflammasome assembly and TRX oxidation. TRX oxidations leads to release of TXNIP, which binds NLRP3 and promotes inflammasome assembly. Inflammasome activation leads to further activation of caspase-1, which cleaves GSDMD and Pro-IL-1β. N-GSDMD oligomerizes in the plasma membrane to form pores, so that proinflammatory IL-1β can be released. ADP = adenosine diphosphate; ATP = adenosine triphosphate; Cyt c = cytochrome c; ETC = electron transport chain; GSDMD = gasdermin D; IL-1β = interleukin 1β; N–GSDMD = N-terminal gasdermin D; NLRP3 = NLR family pyrin domain containing 3; NOX = NADPH oxidase; Pro-IL-1β = Pro-interleukin 1β; RAC = RAC GTPase; ROS = reactive oxygen species; TRX_ox._ = oxidized thioredoxin; TXNIP = thioredoxin-interacting protein. This figure was created with BioRender (https://biorender.com); original files available on request.

Pyroptosis is a pro-inflammatory form of regulated necrotic cell death executed by the pore-forming protein gasdermin D. Pyroptosis is typically initiated by the activation of inflammasomes, multi-protein complexes that sense microbial pathogens or danger signals and activate caspase-1, or by non-canonical pathways engaging caspase-4/5 (in human) or caspase-11 (in mouse) [12,197] (Figure 5). Activated caspases cleave gasdermin D, releasing its N-terminal fragment that oligomerizes in the plasma membrane to form pores, thereby causing cell swelling, membrane rupture, and release of pro-inflammatory mediators such as IL-1β and IL-18 [12,198]. Morphologically, pyroptosis resembles necrosis, but functionally it couples cell death with innate immune signaling, thus playing a central role in host defense and inflammatory pathology [199,200].

As inflammasome activation is the key upstream event in pyroptosis, redox signals have emerged as important modulators of this process. In particular, ROS derived from mitochondria and NOX have been shown to promote NLRP3 inflammasome assembly, thereby linking oxidative stress to the activation of caspase-1 and pyroptotic cell death [15,89]. One important molecular link between ROS and NLRP3 activation is the thioredoxin-interacting protein (TXNIP). Under basal conditions, TXNIP is bound to reduced TRX, but oxidative stress promotes TRX oxidation and the release of TXNIP, which can then directly bind to NLRP3 and promote inflammasome assembly [201] (Figure 5). This mechanism establishes TXNIP as a redox-sensitive switch that couples oxidative imbalance to caspase-1 activation and pyroptosis. Beyond inflammasome regulation, TXNIP plays a broader role in cellular stress responses, as shown by its ability to control oxidative DNA damage and aging through redox-dependent mechanisms [202]. Together, these findings highlight TXNIP as a critical mediator at the interface of ROS signaling, inflammasome activation, and stress-induced cell fate. Multiple intracellular sources of ROS contribute to inflammasome activation and thereby modulate pyroptosis. Mitochondrial ROS are considered a major driver, as mitochondrial dysfunction, mtDNA release, and increased ROS production synergistically promote NLRP3 activation [88]. In addition, NOX-derived ROS have been implicated in priming and activating inflammasomes in phagocytes, linking microbial recognition to oxidative burst responses [203]. Peroxisomes also contribute to the redox balance by generating hydrogen peroxide, which can act as a cofactor in inflammasome signaling under stress conditions [15]. Importantly, the relative contribution of these ROS sources is context-dependent: in some systems, mitochondrial ROS are indispensable for NLRP3 activation, whereas in others NOX activity is dominant, reflecting cell-type and stimulus-specific requirements. This variability underscores the versatility of ROS as modulators of pyroptosis and the complexity of redox–inflammasome crosstalk. Thus, ROS function as critical modulators of pyroptosis by integrating signals from diverse cellular sources to regulate inflammasome activation and shape the balance between host defense and inflammatory pathology. The context-dependent roles of ROS in pyroptosis across different regulatory levels are summarized in Table 4.

### 4.6. ROS as Modulators of Paraptosis

Paraptosis is a non-apoptotic form of programmed cell death first described by Sperandio and colleagues, characterized by extensive cytoplasmic vacuolization originating from swelling of the endoplasmic reticulum (ER) and mitochondria, but lacking nuclear condensation and apoptotic body formation [6,207]. Unlike apoptosis and necroptosis, paraptosis proceeds independently of caspases and is not prevented by classical caspase inhibitors. Its execution often requires sustained activation of mitogen-activated protein kinase (MAPK) pathways, particularly ERK and JNK, and is associated with perturbations of protein homeostasis and ER stress [208]. Due to these features, paraptosis is considered a distinct, morphologically and mechanistically characterized mode of regulated cell death, which has the potential to be relevant in situations where cells resist apoptosis.

Beyond its defining morphological and signaling features, paraptosis has increasingly been linked to disturbances in redox homeostasis. Several studies demonstrate that excessive production of ROS can trigger or amplify paraptotic cell death, particularly by promoting mitochondrial dysfunction and endoplasmic reticulum stress, which are central hallmarks of this pathway [209,210]. These findings suggest that ROS do not merely accompany paraptosis but can actively contribute to its initiation and progression, placing oxidative stress as an important modulator of this non-apoptotic cell death program.

Mechanistically, ROS act as proximal triggers and amplifiers of paraptosis, largely by coupling mitochondrial dysfunction to ER stress. In breast cancer cells, curcumin induces paraptosis in an ROS-dependent manner: mitochondrial superoxide accumulates early, and ROS scavenging markedly reduces ER/mitochondrial vacuolization, highlighting ROS as an initiating signal [209]. Similar mechanisms have been described in recent studies and reviews of paraptosis, where ROS accumulation, ER and mitochondrial dilation, and vacuolization are highlighted as key upstream events [211]. Beyond curcuminoids, δ-tocotrienol has been shown to induce paraptosis in melanoma cells through Ca^2+^ overload and ROS-associated mitochondrial depolarization; antioxidant pretreatment effectively rescues the phenotype, confirming a causal role for ROS [210]. Further evidence comes from jolkinolide B, which triggers paraptosis in bladder cancer cells by inhibiting TrxR1, depleting GSH, and driving ROS-dependent ER stress and ERK activation; these effects are reversed by ROS scavenging [212]. Despite the accumulating evidence from pharmacological models, the physiological relevance of ROS-driven paraptosis remains uncertain. To date, most studies have been conducted in cancer cell lines exposed to natural compounds or synthetic agents, where ROS generation, mitochondrial dysfunction, and ER stress converge to induce paraptotic death [209,210,211,212]. Paraptosis-like morphology has been observed in developmental and neurodegenerative contexts [207,208]. However, ROS-dependent paraptosis has so far only been convincingly demonstrated in pharmacological in vitro models, with its occurrence under physiological conditions remaining uncertain. The role of ROS in paraptosis therefore remains incompletely defined, and further studies are required to clarify whether ROS-dependent mechanisms contribute to this death pathway under physiological or pathological conditions. Importantly, the current evidence for ROS involvement in paraptosis largely relies on pharmacological inducers, and genetic approaches will be essential to establish causality more rigorously [209,210,211,212].

### 4.7. ROS as Modulators of Parthanatos

Parthanatos is a distinct form of regulated cell death mediated by overactivation of poly(ADP-ribose) polymerase-1 (PARP-1) in response to extensive DNA damage, often triggered by oxidative or nitrosative stress. Unlike apoptosis or necroptosis, parthanatos is characterized by the accumulation of poly(ADP-ribose) (PAR) polymers, which act as toxic messengers to propagate cell death signals. Excessive PARP-1 activity depletes cellular NAD^+^ and ATP pools, thereby impairing energy metabolism, but most critically generates PAR polymers that translocate from the nucleus to mitochondria. There, PAR binds to apoptosis-inducing factor (AIF) and promotes its release. Once liberated, AIF translocates to the nucleus, where it induces DNA fragmentation-mediated cell death [213,214,215].

ROS modulate parthanatos primarily by determining the threshold at which DNA damage shifts from repairable lesions to lethal signaling. Moderate ROS levels activate DNA repair without triggering cell death, whereas excessive ROS generate clustered DNA strand breaks that hyperactivate PARP-1. This disproportionate PARP-1 response results in massive synthesis of PAR polymers, which act as death signals by binding to AIF and promoting its nuclear translocation [213,214,215]. In the nucleus, AIF drives large-scale DNA fragmentation and chromatin condensation in a caspase-independent manner. Unlike the oligonucleosomal fragmentation typical of apoptosis, AIF induces extensive, ~50-kb DNA fragmentation, leading to a catastrophic loss of genomic integrity [213,216]. Mechanistically, AIF promotes chromatinolysis by directly interacting with the H2A histone family member X (H2AX), thereby initiating caspase-independent programmed necrosis [217]. In addition, the macrophage migration inhibitory factor (MIF) has been identified as the nuclease activity required for AIF-dependent DNA degradation. Upon PARP-1 hyperactivation, PAR polymers facilitate AIF–MIF complex formation, and the nuclease activity of MIF executes the large-scale DNA cleavage characteristic of parthanatos [218]. Importantly, ROS also sustain parthanatos through feed-forward loops. PARP-1 hyperactivation and mitochondrial dysfunction enhance mitochondrial ROS production, which in turn aggravates DNA damage and further stimulates PARP-1 activity. This vicious cycle amplifies PAR accumulation and accelerates large-scale DNA fragmentation, thereby ensuring that once initiated, parthanatos proceeds irreversibly to cell death [219,220,221,222]. Together, these findings underscore that ROS not only initiate but also perpetuate parthanatos, positioning oxidative stress as both the trigger and the driving force that determines the inevitability of this unique form of RCD.

### 4.8. ROS as Modulators of Lysosome-Dependent Cell Death

Lysosome-dependent cell death (LDCD) is a form of regulated cell death initiated by lysosomal membrane permeabilization (LMP). When LMP occurs, lysosomal hydrolases, especially cathepsins, enter the cytosol. Limited LMP typically engages apoptotic signaling (e.g., cathepsin-mediated BID cleavage, MOMP, caspase activation), whereas widespread lysosomal rupture can precipitate rapid, caspase-independent cell death. The cellular outcome depends on the magnitude of LMP, lysosomal enzyme repertoire, and mitochondrial crosstalk, placing lysosomes at a central decision point in cell fate control [21,223]. To counteract lysosomal destabilization, cells activate protective mechanisms such as the endosomal sorting complexes required for transport (ESCRT) machinery, which rapidly repairs small lysosomal lesions, and lysophagy, the selective autophagic removal of irreparably damaged lysosomes. ESCRT components are recruited to damaged membranes (in part via Ca^2+^-sensitive adaptors), restoring integrity; when repair fails, damaged lysosomes are tagged (e.g., by galectin binding and ubiquitination) and cleared by lysophagy to preserve homeostasis. Failure of these defenses shifts the balance toward cell death and pathology, underscoring lysosomes as key regulators at the interface of survival and death [224,225,226,227].

ROS promote LDCD primarily through intra-lysosomal Fenton chemistry, in which H_2_O_2_ reacts with labile Fe^2+^ to generate •OH. These highly reactive •OH species initiate lipid peroxidation of the lysosomal membrane, leading to destabilization and the release of cathepsins and other hydrolases [228,229]. Lysosomes, as major sites of iron storage and turnover, are particularly vulnerable to this process. Experimentally, intra-lysosomal ROS accumulation induces LMP and cathepsin D release. Whereas lysosomotropic iron chelators such as desferrioxamine (DFO) prevent these events [230]. The outcome of ROS-induced LMP depends on its extent. Partial and limited LMP releases small amounts of cathepsins, which cleave BID to tBID, thereby activating BAX/BAK, inducing MOMP, cytochrome c release, and caspase-dependent apoptosis [21,223,231]. In contrast, extensive LMP leads to massive leakage of cathepsins and other hydrolases into the cytosol, overwhelming mitochondrial signaling and degrading essential cellular proteins directly. This results in rapid loss of membrane integrity and caspase-independent necrotic cell death [21,223]. Thus, ROS act as critical modulators of LDCD, where moderate levels promote apoptotic signaling via controlled LMP, while excessive ROS drive catastrophic lysosomal rupture and necrotic cell death (Table 5).

### 4.9. ROS as Modulators of Oxeiptosis

Oxeiptosis is a regulated, caspase-independent cell death pathway mediated by the Kelch-like ECH-associated protein 1 (KEAP1)/phosphoglycerate mutase family member 5 (PGAM5)/the apoptosis-inducing factor mitochondria-associated 1 (AIFM1) axis. In this process, PGAM5, a mitochondrial phosphatase, dephosphorylates and activates AIFM1, which then translocates to the nucleus and induces large-scale chromatin condensation and DNA fragmentation. Unlike necroptotic cell death, oxeiptosis does not involve plasma membrane rupture and therefore avoids the uncontrolled release of damage-associated molecular patterns (DAMPs). As a result, oxeiptosis is considered an apoptosis-like caspase-independent cell death pathway that serves as a safeguard mechanism to eliminate stressed cells without triggering inflammation [23].

While oxeiptosis is defined by the KEAP1–PGAM5–AIFM1 axis, its initiation is tightly linked to oxidative stress. Excessive ROS oxidize critical cysteine residues within KEAP1, impairing its canonical role in targeting NRF2 for proteasomal degradation. Under these conditions, KEAP1 is redirected to interact with mitochondrial PGAM5, which dephosphorylates and activates AIFM1, thereby driving nuclear translocation and large-scale DNA fragmentation. In this way, ROS act as upstream signals that convert KEAP1 from a sensor of redox homeostasis into a trigger of the oxeiptotic death program, ensuring that cells with irreparable oxidative damage are removed in a non-inflammatory manner [23].

### 4.10. ROS as Modulators of NETosis

NETosis is a specialized form of regulated neutrophil cell death characterized by the release of neutrophil extracellular traps (NETs). These web-like structures consist of decondensed chromatin decorated with histones and granular proteins such as neutrophil elastase (NE) and myeloperoxidase (MPO). NETs function as antimicrobial barriers that immobilize and neutralize invading pathogens, thereby complementing phagocytosis and degranulation. In addition to this “suicidal” form, neutrophils can also release NETs through vital NETosis, a process in which they remain viable and functionally active after NET extrusion. Beyond host defense, dysregulated NET formation has been implicated in sterile inflammation, autoimmunity, and thrombotic disorders, highlighting its dual role as a protective and potentially pathogenic process [232,233,234].

In suicidal NETosis, a burst of NOX2-derived ROS is required; neutrophils from patients with chronic granulomatous disease fail to release NETs [90]. Downstream of ROS, NE and MPO translocate from azurophilic (primary) granules, lysosome-like organelles enriched in antimicrobial enzymes, into the nucleus, where they promote chromatin decondensation: NE via degrading histones and MPO by cooperating in chromatin remodeling [235,236]. In parallel, the enzyme peptidylarginine deiminase 4 (PAD4) modifies histones by converting arginine residues to citrulline. This reduces the electrostatic interaction between histones and DNA, loosening chromatin structure and enabling efficient NET release. Consistently, loss of PAD4 function strongly diminishes NET formation and compromises antimicrobial defense [237].

Beyond NOX2, mitochondria provide an additional source of ROS that can drive NET formation under certain stimuli. In this context, so-called vital NETosis describes a rapid form of NET release in which neutrophils extrude NETs without undergoing immediate cell death, thereby remaining viable and functionally active in antimicrobial defense. This process often depends on mitochondrial ROS and calcium influx, and contrasts with suicidal NETosis, where the neutrophil ultimately dies [234]. Thus, both NOX2-dependent and mitochondrial ROS act as key modulators of NETosis, with their relative contribution depending on the type and strength of stimulation. However, accumulating evidence indicates that NET release can also occur in an ROS-independent manner, particularly in response to physiological agonists such as calcium ionophores or monosodium urate crystals [238,239,240]. These conflicting findings suggest that ROS requirements in NETosis are stimulus-specific rather than universal and may vary according to neutrophil state, extracellular milieu, and assay readouts. Consequently, the role of ROS in NETosis should be regarded as context-dependent, ranging from indispensable initiators in canonical suicidal NETosis to modulators or dispensable factors in alternative NET-releasing pathways [90,241]. Taken together, ROS emerge as central regulators of NETosis, determining whether neutrophils undergo suicidal death with NET release or generate NETs in a vital manner, thereby linking redox balance to both host defense and inflammatory pathology (Table 6).

### 4.11. ROS as Modulators of Autophagy

**Figure 6:**
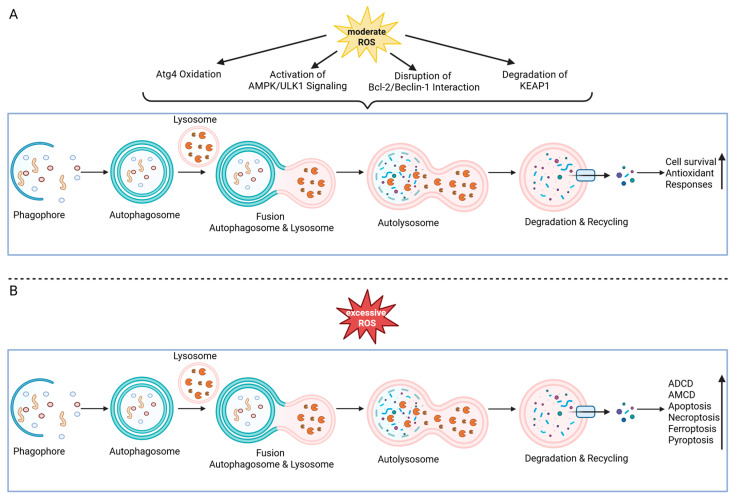
Autophagy. (**A**) Moderate ROS concentrations induce autophagy through multiple mechanisms, including oxidation and inactivation of autophagy-related protein 4 (Atg4), activation of AMP-activated protein kinase (AMPK)/Unc-51-like kinase 1 (ULK1) signaling, disruption of the B-cell lymphoma 2 (Bcl-2)/Beclin-1 interaction, and autophagic degradation of Kelch-like ECH-associated protein 1 (KEAP1). Autophagy is a lysosome-directed pathway in which the fusion of an autophagosome with a lysosome generates an autolysosome. The degradation of damaged organelles and protein aggregates helps to maintain tissue homeostasis, promote cell survival, and support antioxidant responses. (**B**) Excessive ROS shift autophagy from a cytoprotective process to a dysregulated, lethal form that directly contributes to cell death. Uncontrolled autophagy can promote cell death by degrading essential cellular components and organelles, a process referred to as autophagy-dependent cell death (ADCD), also known as autophagy-mediated cell death (AMCD). Moreover, it intersects with other cell death pathways, including apoptosis, necroptosis, ferroptosis, and pyroptosis. ADCD = autophagy-dependent cell death; AMCD = autophagy-mediated cell death; AMPK/ULK1 = AMP-activated protein kinase/Unc-51-like kinase 1; Atg4 = autophagy-related protease 4; Bcl-2 = B-cell lymphoma 2; KEAP1 = kelch-like ECH-associated protein 1; ROS = reactive oxygen species. This figure was created with the assistance of BioRender (https://biorender.com); original files available on request.

Autophagy is a conserved lysosome-directed pathway that engulfs cytoplasmic material, including damaged organelles and protein aggregates, into autophagosomes for degradation and recycling, thereby maintaining intracellular quality control and tissue homeostasis [242,243,244]. Under nutrient limitation or energetic stress, autophagy is rapidly induced to provide metabolic substrates (amino acids, lipids, nucleosides) that sustain ATP production and biosynthesis, bridging transient energy shortages and promoting survival [245,246] (Figure 6A). Although fundamentally adaptive, when stress is severe or prolonged, autophagy can become over-activated or re-purposed and contribute to programmed cell death. In this context, autophagy not only operates as a cell-death mechanism in its own right but also intersects with other death pathways, including apoptosis, via shared regulators such as Bcl-2 family proteins and Beclin-1 [247,248], necroptosis, through modulation of RIPK1/RIPK3 signaling [249], ferroptosis via ferritinophagy and increased iron availability [250], and pyroptosis by regulating inflammasome activation (e.g., NLRP3) and associated mechanisms [251,252] (Figure 6B).

ROS—not only byproducts of metabolism but also potent signaling molecules—play a dual role in modulating autophagy. Under moderate oxidative stress, ROS can activate autophagy through multiple mechanisms, including oxidation and inactivation of autophagy-related protease 4 (Atg4), activation of AMP-activated protein kinase (AMPK)/Unc-51-like kinase 1 (ULK1) signaling, disruption of the Bcl-2/Beclin-1 interaction, and the autophagic degradation of Kelch-like ECH-associated protein 1 (KEAP1). Thereby Nrf2-mediated antioxidant responses are enhanced and survival is promoted [253,254] (Figure 6A). Conversely, autophagy serves as a negative feedback mechanism, selectively removing damaged, ROS-producing mitochondria via mitophagy or peroxisomes via pexophagy, thus mitigating oxidative stress and maintaining cellular redox homeostasis [253]. However, when ROS accumulation is excessive or prolonged, autophagy may shift from a cytoprotective process to a dysregulated, lethal form that directly contributes to cell death (Figure 6B). In transformed and cancer cells, sustained ROS exposure induces autophagic cell death, and inhibition of autophagy confers protection even in the absence of apoptosis, highlighting autophagy as a distinct death mechanism [255,256]. More broadly, excessive or uncontrolled autophagy can promote cell death by indiscriminate degradation of essential cellular components and organelles, a process defined as autophagy-dependent cell death (ADCD) or autophagy-mediated cell death (AMCD) [257,258].

Nevertheless, the necessity of ROS for autophagy initiation remains controversial. Pioneering studies demonstrated that starvation-induced autophagy requires ROS-dependent oxidation of ATG4 [259,260], establishing redox signals as key triggers of autophagosome formation. In contrast, pharmacological inhibition of mTOR with rapamycin reliably induces autophagy in an ROS-independent manner, and in vivo antioxidant treatment can fail to suppress fasting-induced autophagy [261,262]. These discrepancies likely reflect differences in stimuli (starvation versus mTOR blockade), basal redox buffering, and the relative contribution of mitochondrial or peroxisomal ROS. Thus, while ROS clearly shape the amplitude and quality of autophagy, they should be considered context-dependent modulators rather than universal initiators [263,264] (Table 7). This dual role highlights the context-dependent nature of the ROS–autophagy axis: at low to moderate levels, ROS induce protective autophagy that supports cell survival, whereas sustained or high ROS levels trigger destructive autophagy that contributes to cell death [254,265].

### 4.12. ROS and Other Forms of Cell Death

In addition to the cell death pathways discussed in detail in the preceding chapters, ROS also influence several other forms of regulated cell death, which will be briefly outlined in the following section. In total, we provide in-depth discussion of eleven modalities (apoptosis, anoikis, necroptosis, ferroptosis, pyroptosis, paraptosis, parthanatos, lysosome-dependent cell death, both suicidal and vital NETosis and autophagy-dependent cell death), as these are supported by the most extensive mechanistic evidence for ROS involvement. By contrast, other RCD modalities such as entosis, mitotic catastrophe, mitotic death, cuproptosis, alkaliptosis, methuosis, and disulfidptosis are less well characterized, with current insights into ROS regulation largely limited to isolated reports or pharmacological models. We therefore summarize these pathways more briefly, highlighting their emerging links to redox biology while acknowledging that the field is still developing. This approach ensures proportional coverage that reflects the strength of available evidence in the literature, while still providing a comprehensive overview across 19 modalities.

#### 4.12.1. ROS and Mitochondrial Permeability Transition-Driven Necrosis

Mitochondrial permeability transition (MPT)-driven necrosis is a regulated form of necrotic cell death caused by sustained opening of the mitochondrial permeability transition pore (mPTP) in the inner mitochondrial membrane. Triggered by calcium (Ca^2+^) overload and oxidative stress, pore opening leads to mitochondrial swelling, loss of membrane potential, and bioenergetic collapse [266,267]. ROS act as both inducers and amplifiers of this process: they directly promote mPTP opening and drive a vicious cycle known as ROS-induced ROS release (RIRR), in which mitochondrial dysfunction further enhances ROS production. This self-amplifying loop irreversibly collapses mitochondrial function, depletes ATP, and propagates oxidative damage to macromolecules, thereby shifting the cell from a potentially reversible stress response into necrotic cell death [268,269].

#### 4.12.2. ROS and Entotic Cell Death

Entotic cell death is a distinctive form of RCD often referred to as a type of “cellular cannibalism”. A living cell actively invades a neighboring cell, creating a “cell-in-cell” structure. The internalized cell is subsequently degraded by the host cell through lysosomal pathways, involving LC3-associated phagocytosis (LAP), autophagy-related proteins such as the autophagy related (ATG) 5, ATG7, the class III PI 3-kinase VPS34, and cathepsin-mediated digestion, independently of caspase activation [25,26,270]. ROS, particularly those generated by NOX2, facilitate entosis by promoting LC3 lipidation at the vacuolar membrane, a critical step in LAP required for lysosomal clearance of the internalized cell [26,271,272]. Thus, ROS may act as modulators of entosis by supporting the autophagy-like degradation process within the host cell.

#### 4.12.3. ROS and Mitotic Catastrophe

Mitotic catastrophe (MC) is a tumor-suppressive fail-safe triggered by defective mitosis (e.g., DNA damage, spindle/checkpoint failure) that channels genomically unstable cells into cell death or permanent arrest. Morphologically it features multinucleation and micronuclei and typically culminates in apoptosis, necrosis, or senescence [273,274]. ROS act as both inducers and modulators of MC. Elevated ROS cause oxidative DNA lesions and spindle damage, thereby increasing the likelihood of aberrant mitosis [275,276]. ROS also activate cell cycle checkpoints through redox activation of ataxia telangiectasia mutated (ATM) and ataxia telangiectasia and Rad3-related (ATR)/checkpoint kinase 1 (CHK1) signaling, enforcing G_2_/M control. ATM can be activated by oxidation even without DNA double-strand breaks (DSBs), while hyperoxia and H_2_O_2_ stimulate ATR/CHK1, with apurinic/apyrimidinic endonuclease 2 (APE2) required for H_2_O_2_-induced CHK1 activation [277,278,279]. Downstream, CHK1/CHK2 inhibit cell division cycle 25C (CDC25C) via phosphorylation, and ROS can directly oxidize or degrade CDC25C, both of which prevent CDK1/cyclin B activation and maintain arrest [280,281,282]. Under persistent or excessive ROS, these checkpoint barriers collapse and cells undergo MC, which represents an upstream safeguard rather than a terminal RCD, since cell elimination occurs indirectly via apoptosis, necrosis, or senescence [274].

#### 4.12.4. ROS and Mitotic Death

In contrast, mitotic death (MD) constitutes a bona fide form of regulated cell death that is executed directly during or immediately after defective mitosis. While MC is a fail-safe defined by aberrant mitotic progression and morphological hallmarks such as multinucleation, MD represents the terminal death program itself. ROS accumulation represents a prominent driver of MD, peaking during mitosis and enhancing DNA and protein damage under conditions of mitotic arrest. Elevated ROS further promote mitochondrial dysfunction and activation of intrinsic apoptotic signaling, thereby driving MD as a discrete RCD entity mechanistically and conceptually distinct from MC [94,283,284,285].

#### 4.12.5. ROS and Cuproptosis

Cuproptosis is a copper-dependent, non-apoptotic form of RCD, mechanistically distinct from apoptosis, ferroptosis, and pyroptosis. It is triggered by excess intracellular Cu^2+^ binding directly to lipoylated mitochondrial enzymes within the tricarboxylic acid cycle (TCA), leading to their aggregation, destabilization of iron–sulfur (Fe–S) cluster proteins, and proteotoxic stress [286,287,288]. Although ROS generation is not the primary trigger, copper overload fosters redox activity. Elevated ROS arise from copper-mediated oxidative reactions and Fe–S cluster impairment, amplifying mitochondrial damage and reinforcing the death process [288,289]. Thus, ROS in cuproptosis act as downstream amplifiers of cell death, rather than initial inducers, distinguishing its oxidative dimension from the lipid-peroxide-driven ferroptosis.

#### 4.12.6. ROS and Alkaliptosis

Alkaliptosis is a pH-dependent form of cell death, characterized by intracellular alkalinization and caspase independence. It differs from apoptosis, ferroptosis, or pyroptosis because its execution is governed by disruption of pH homeostasis and lysosomal function, rather than protease activation, lipid peroxidation, or pore formation [290]. The involvement of ROS in alkaliptosis is less clearly defined than in other RCD modalities. Alkalinization can promote oxidative stress and mitochondrial dysfunction, but current evidence suggests ROS act primarily as secondary amplifiers rather than as initiating triggers of the pathway [290,291].

#### 4.12.7. ROS and Methuosis

Methuosis is a nonapoptotic form of regulated cell death first recognized in Ras-transformed glioblastoma cells, characterized by pronounced cytoplasmic vacuolization. It results from hyperactivated macropinocytosis: excessive fluid uptake generates large, single-membrane vacuoles (derived from macropinosomes) that fail to acidify, recycle, or fuse with lysosomes, instead accumulating and coalescing until they compromise cellular integrity, leading to swelling, metabolic failure, and plasma membrane rupture [28,292,293,294,295]. Methuosis is driven by Ras/Ras-related C3 botulinum toxin substrate 1 (Rac1) signaling with impaired ADP-ribosylation factor 6 (Arf6) recycling but can also be induced by small molecules such as the indole-chalcone 3-(5-methoxy-2-methyl-1H-indol-3-yl)-1-(4-pyridinyl)-2-propen-1-one (MOMIPP). MOMIPP inhibits the FYVE finger-containing phosphoinositide kinase (PIKfyve), leading to vacuole accumulation [294,296,297], and concurrently activates the stress kinase c-Jun N-terminal kinase (JNK1/2), a redox-sensitive pathway [298]. Therefore, although redox-sensitive pathways may accompany methuosis, a causal role for ROS is unproven and remains unresolved. To date, most insights into ROS involvement during methuosis stem from studies with small-molecule inducers such as MOMIPP, which may exert pleiotropic effects beyond vacuolization [294,296,297,298]. Therefore, genetic approaches targeting redox regulators will be required to clarify whether ROS act as bona fide drivers of methuotic cell death.

#### 4.12.8. ROS and Disulfidptosis

Disulfidptosis is a recently identified form of RCD first reported in 2023 [299], triggered by the accumulation of abnormal disulfide bonds in cytoskeletal proteins under conditions of glucose starvation and high cystine uptake. This process leads to actin cytoskeleton collapse and cell death, distinguishing it from apoptosis, ferroptosis, or necroptosis, which rely on caspase activation, lipid peroxidation, or RIPK signaling, respectively. The contribution of ROS remains under investigation: although oxidative stress may exacerbate disulfide stress, current evidence indicates that disulfide bond accumulation itself, rather than ROS, is the primary driver of this pathway [299,300,301]. While these initial studies provide important mechanistic insights, the link between ROS and disulfidptosis is largely indirect. Much of the current evidence relies on pharmacological or metabolic perturbations, where ROS may accumulate secondarily due to glucose deprivation and cystine uptake rather than acting as direct effectors of cell death [299,300,301]. Thus, it remains unclear whether ROS play a causal role in amplifying disulfide stress or represent a by-product of metabolic collapse. Future studies employing genetic approaches, such as modulation of cystine transporters, thiol–disulfide oxidoreductases, or ROS-detoxifying enzymes, will be essential to establish causality. Such strategies will help to determine whether ROS function merely as ancillary stress signals or as bona fide drivers of the disulfidptotic process under physiological or pathological conditions.

Across the 19 RCD modalities reviewed, ROS act in most as context-dependent initiators, modulators, and amplifiers, positioning redox control as a central determinant of cell-fate decisions.

## 5. ROS-Driven Crosstalk Between RCD Pathways

ROS orchestrate crosstalk and switching between RCD pathways. Oxidative inhibition of caspase-8 shifts TNF signaling away from apoptosis toward necroptosis, in which ROS further reinforce RIPK1/RIPK3/MLKL-dependent cell death [142,143,144,168,302]. In paraptosis, ROS exacerbate ER/mitochondrial stress and cytoplasmic vacuolation, reinforcing this caspase-independent death program, which is frequently considered an alternative route of cell death when apoptosis is blocked [6]. For lysosome-dependent cell death, ROS are potent triggers of LMP. When LMP is limited and partial, small amounts of cathepsins leak into the cytosol and cleave Bid, thereby engaging MOMP, cytochrome c release, and apoptosis. In contrast, extensive LMP causes massive release of lysosomal hydrolases, overwhelming cellular proteostasis and leading to necrosis-like cell death [21]. In ferroptosis, iron-dependent lipid peroxidation executes a caspase-independent death program; mitochondria characteristically appear shrunken with condensed membranes and reduced cristae. Beyond its unique lipid-ROS signature, ferroptosis intersects with other RCDs. p53-mediated repression of SLC7A11 links stress responses to ferroptotic signaling and can divert cell fate away from apoptosis when cystine/GSH defenses collapse. In addition, ferritinophagy (NCOA4-mediated) connects autophagy to ferroptosis by liberating redox-active iron that fuels lipid peroxidation. Finally, ferroptotic cells release High mobility group box 1 (HMGB1) as a DAMP, propagating inflammatory signaling and functionally bridging ferroptosis to immune-driven death programs [193,250,303]. Pyroptosis also engages in ROS-mediated crosstalk with other RCD pathways. Oxidative stress activates the NLRP3 inflammasome, serving as a crucial link between elevated ROS levels and the execution of pyroptotic cell death [304]. Moreover, DAMPs like HMGB1, released during ferroptosis, amplify inflammasome signaling and can promote pyroptosis in neighboring immune cells [303]. Parthanatos is not only initiated by ROS, ROS also position it at the intersection of other death programs: when caspases are active, their PARP1 cleavage favors classical apoptosis, whereas caspase inhibition or energy collapse permits sustained PARP1 activity and shifts fate toward parthanatos; conversely, PARP1 overactivation can suppress apoptotic execution [305]. Crosstalk with necroptosis and ferroptosis arises because ROS channel damage toward different molecular targets—nuclear DNA (driving parthanatos) versus peroxidizable membrane lipids (driving ferroptosis)—so that redox localization and antioxidant capacity determine which RCD predominates [306]. In addition, ROS mediate critical crosstalk between MPT-driven necrosis and other RCD modalities. Mitochondrial ROS can enhance apoptosis by promoting the release of cytochrome c and facilitating classical apoptotic cascades. However, when caspase activity is impaired, the same oxidative conditions favor necrotic collapse through uncontrolled MPT opening. Concurrently, ROS produced downstream of RIPK3/MLKL activation in necroptosis reinforce MPT-dependent necrosis, blurring the boundaries between these necrotic pathways. Furthermore, mitochondrial ROS-mediated lipid peroxidation can drive or amplify ferroptosis, linking mitochondrial stress to iron-dependent, lipid-ROS-driven death. Together, these interactions emphasize how the localization and intensity of ROS can decisively shift cells between apoptosis, necroptosis, MPT-driven necrosis and ferroptosis [307]. ROS critically influence the fate of cells undergoing aberrant mitosis. Elevated oxidative stress during mitotic arrest leads to the accumulation of oxidized proteins thereby promoting mitotic catastrophe as an oncosuppressive mechanism. When apoptotic machinery remains functional, ROS amplify intrinsic apoptosis as the dominant route of mitotic death, whereas disruption of caspase activity can divert outcomes toward necrotic or senescent paths. In this way, ROS act as molecular selectors, determining whether mitotic failure culminates in mitotic catastrophe, apoptosis, necrosis, or senescence [283].

**Figure 7:**
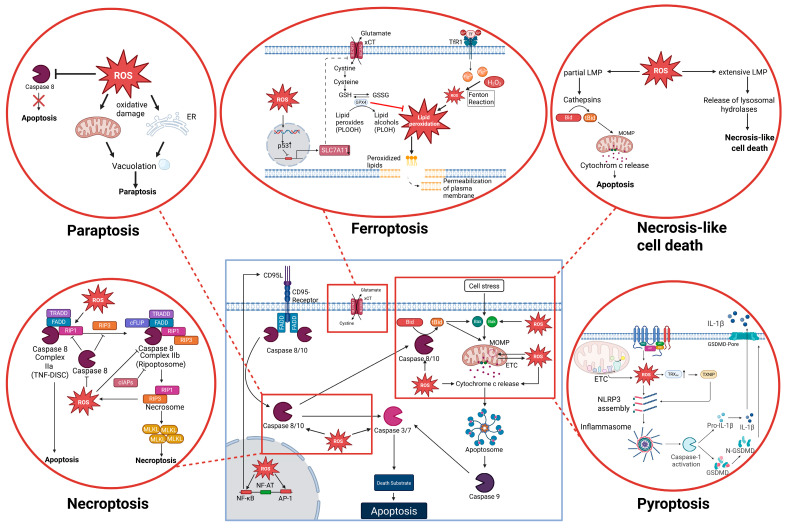
Crosstalk between main RCD pathways. Crosstalk between apoptosis, necroptosis, paraptosis, ferroptosis, necrosis-like cell death and pyroptosis. Oxidative inhibition of caspase-8 shifts TNF signaling from apoptosis toward necroptosis, where ROS further reinforce RIPK1/RIPK3/MLKL-dependent cell death. When apoptosis is blocked, oxidative damage induces ER and mitochondrial stress as well as cytoplasmic vacuolation, leading to paraptosis. ROS also promote extensive lysosomal membrane permeabilization (LMP) and the release of lysosomal hydrolases, resulting in necrosis-like cell death. Ferroptosis is triggered through Fenton-reaction-driven lipid peroxidation, which compromises plasma membrane integrity. Crosstalk also occurs with pyroptosis, where ROS generated by the ETC and NOX complexes promote inflammasome assembly and activation of caspase-1, leading to cleavage of pro-IL-1β and gasdermin. ADP = adenosine diphosphate; AP-1 = activator protein 1; ATP = adenosine triphosphate; Bak = Bcl-2 homologous antagonist/killer; Bax = Bcl-2-associated X protein; Bid = BH3 interacting domain death agonist; CD95L = CD95 (Fas) ligand; cIAPs = cellular inhibitor of apoptosis proteins; cFLIP = FLICE-like inhibitory protein; Cyt c = cytochrome c; ER = endoplasmatic reticulum; ETC = electron transport chain; FADD = Fas-associating death domain-containing protein; Fe^2+^ = ferrous; Fe^3+^ = ferric; GSDMD = gasdermin D; GPX4 = phospholipid hydroperoxide glutathione peroxidase 4; GSH = reduced glutathione; GSSG = oxidized glutathione; H_2_O_2_ = hydrogen peroxide; IL-1β = interleukin 1β; LMP = lysosomal membrane permeabilization; MLKL = mixed lineage kinase domain like pseudokinase; NF-AT = nuclear factor of activated T cells; NF-κB = nuclear factor kappa-light-chain-enhancer of activated B cells; N—GSDMD = N-terminal gasdermin D; NLRP3 = NLR family pyrin domain containing 3; p53 = transcription factor p53; PLOH = lipid alcohols; PLOOH = lipid peroxides; Pro-IL-1β = Pro-interleukin 1β; RAC = RAC GTPase; RIP1/3 = receptor-interacting protein kinase 1/3; ROS = reactive oxygen species; SLC3A2 = heavy chain of xCT antiporter; SLC7A11 = light chain of xCT antiporter; tBID = truncated BH3 interacting domain death agonist; Tf = transferrin; TfR1 = transferrin receptor 1; TRADD = TNF receptor type 1-associated death domain protein; TRX_ox._ = oxidized thioredoxin; TXNIP = thioredoxin-interacting protein; xCT = cystine/glutamate antiporter system. This figure was created with the assistance of BioRender (https://biorender.com); original files available on request.

Together, these interactions emphasize how the localization and intensity of ROS can decisively shift cells between apoptosis, necroptosis, MPT-driven necrosis, and ferroptosis (Figure 7). This integrative overview illustrates how ROS orchestrate molecular crosstalk among distinct RCD pathways and highlights their context-dependent duality as both signaling mediators and damaging molecules.

## 6. Emerging Technologies, Future Directions, and Challenges in Redox–RCD Research

Recent years have transformed redox biology with tools that resolve where, when, and how ROS shape RCD. Genetically encoded biosensors now quantify organelle-specific oxidant flux in living cells and tissues: HyPer7 enables ultrafast, pH-robust H_2_O_2_ imaging [308], while roGFP-based sensors (Grx1-roGFP2 for glutathione redox; roGFP2-Orp1 or Tsa2ΔCR for peroxiredoxin-coupled H_2_O_2_) provide subcellular precision [309,310]. Optogenetic and chemogenetic perturbations such as KillerRed, SuperNova, or D-amino acid oxidase (DAO) now allow controlled ROS production in specific compartments, enabling causal tests of whether oxidative signals are required to initiate autophagy, NETosis, or ferroptosis [311,312]. At the systems level, genome-wide CRISPR screens have uncovered unexpected regulators of ferroptosis and disulfidptosis, including GPX4/SLC7A11, SWI/SNF–NRF2 signaling, and CRTC3, thereby expanding the map of genetic vulnerabilities [313,314]. Complementary redox chemoproteomic platforms—including OxICAT (Oxidation-dependent Isotope-Coded Affinity Tagging), OxiTMT (oxidation-dependent Tandem Mass Tagging), and dimedone-based sulfenome mapping—permit site-specific quantification of cysteine oxidation, linking ROS bursts to signaling nodes of apoptosis, necroptosis, or autophagy [315,316]. Finally, lipid peroxidation imaging with C11-BODIPY(581/591) or PALP, and spatial lipidomics via MALDI-MSI, provide direct readouts of ferroptosis execution [317,318].

These technologies are expected to resolve long-standing controversies—for example, whether NETosis and autophagy universally require ROS—by enabling stimulus-specific, compartment-resolved, and single-cell analyses. They also open the possibility of integrating biosensors with spatial transcriptomics and intravital microscopy to uncover how ROS-driven death pathways operate within complex tissue microenvironments, including tumors and inflamed organs.

At the same time, important translational limitations must be acknowledged. Much of the evidence in ROS–RCD research derives from pharmacological studies in immortalized cancer cell lines, often at supra-physiological drug concentrations, while genetic validation and in vivo confirmation remain comparatively sparse. Moreover, ROS detection itself is methodologically challenging, as conventional probes lack specificity for distinct species and subcellular pools, complicating interpretation. Finally, the inflammatory consequences of RCD are strongly context-dependent and shaped by the tissue microenvironment, limiting generalization across systems. These caveats should not be viewed as diminishing the significance of current findings but rather as concrete opportunities: by complementing pharmacological data with genetic perturbations, advanced redox imaging, and in vivo validation, the field can progressively bridge mechanistic insight and therapeutic translation.

Together, these emerging methods provide a framework for the mechanistic dissection of ROS–RCD interactions, while emphasizing that methodological rigor and multi-scale integration are essential to reconcile conflicting data and to translate redox biology into therapeutic practice.

## 7. Conclusions

This review synthesizes 19 RCD modalities through the lens of ROS and shows that nearly all are shaped by redox control. In the best-characterized forms of RCDs, including apoptosis, anoikis, necroptosis, ferroptosis, pyroptosis, paraptosis, parthanatos, LDCD, NETosis and autophagy-dependent cell death, ROS act at defined biochemical nodes to initiate, modulate and amplify death signaling, thereby influencing modality selection and inflammatory phenotype. For less frequently described modalities, including cuproptosis, alkaliptosis, methuosis and disulfidptosis, evidence indicates possible ROS involvement; however, causal roles remain incompletely defined and context-specific. Overall, ROS emerge as a unifying yet context-dependent determinant of RCD and a strategic lever for therapeutic intervention. A comparative overview of plasma membrane integrity and inflammatory potential across modalities is provided in Table 8.

ROS modulate initiation and execution thresholds at redox-sensitive RCD checkpoints, thereby influencing pathway selection and the inflammatory phenotype of cell death [319]. By tuning caspase activity and RIPK1/RIPK3/MLKL signaling (apoptosis ↔ necroptosis crosstalk), controlling lipid-peroxidation via the GPX4 axis (ferroptosis), and shaping inflammasome/GSDMD function (pyroptosis), ROS bias cells from immunologically “silent” programs toward pro-inflammatory modalities [320,321,322,323]. This cell death pathway reprogramming has translational implications, including the induction of ferroptosis in refractory tumors and the attenuation of necroptosis or pyroptosis to limit sterile inflammation [324,325,326,327,328].

**Figure 8:**
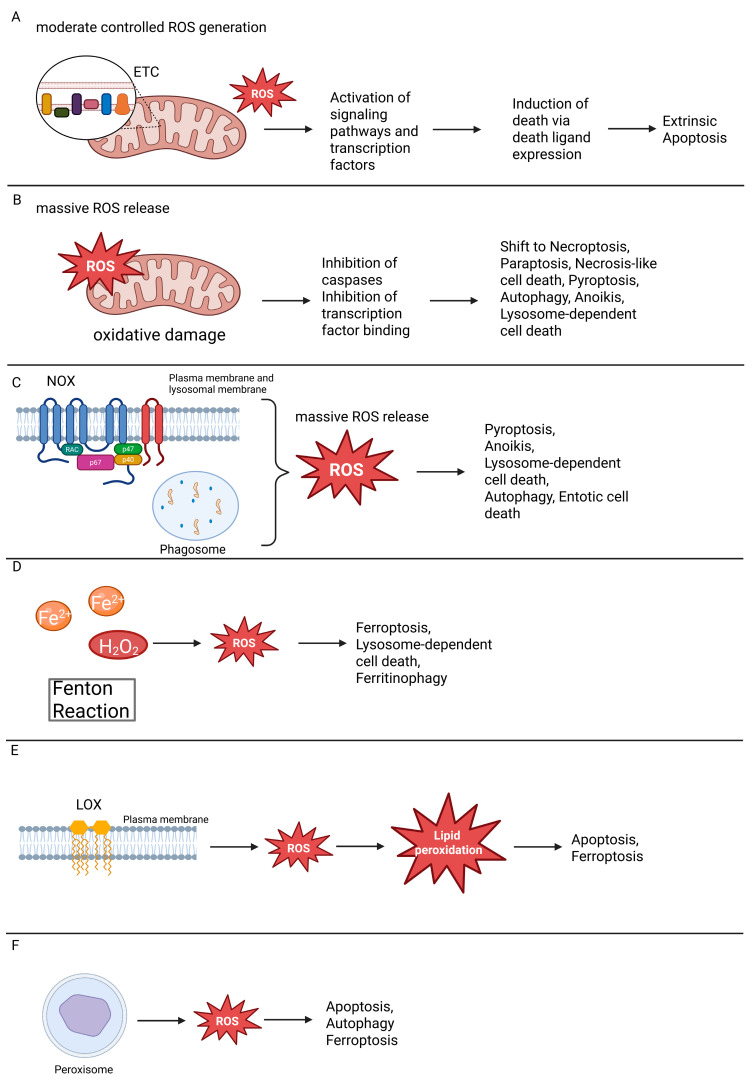
ROS sources and influences on RCDs. (**A**) Moderately controlled ROS generation in mitochondria via the ETC leads to activation of signaling pathways and transcription factors. Activation of these transcription factors induces death receptor ligand expression, promoting extrinsic apoptosis. (**B**) Mitochondrial damage leads to excessive ROS release, which in turn causes further mitochondrial injury and additional ROS production, creating a self-amplifying cycle of oxidative stress. This progressive feedback loop can inhibit caspase activity and transcription factor binding, thereby shifting cell death toward other RCD types such as necroptosis, paraptosis, necrosis-like cell death, pyroptosis, autophagy, anoikis, and lysosome-dependent cell death. (**C**) Other ROS sources, such as phagosomes and NOX complexes, can release high levels of ROS that, if not neutralized by cellular antioxidant defenses, can induce different forms of cell death depending on the type and extent of oxidative damage. The specific outcome—pyroptosis, anoikis, lysosome-dependent cell death, autophagy, or entotic cell death—depends on which cellular structures and pathways are most affected, and is strongly modulated by the efficiency of the oxidative defense system. (**D**) The Fenton reaction occurs when free Fe^2+^ reacts with H_2_O_2_, generating highly reactive hydroxyl radicals (•OH). This process is enhanced under conditions of elevated free iron or impaired antioxidant defenses. ROS generated through this mechanism can promote ferroptosis, lysosome-dependent cell death, and ferritinophagy. (**E**) LOX enzymes generate ROS and initiate the formation of lipid radicals, which can propagate oxidative chain reactions leading to lipid peroxidation of cellular membranes. This process contributes to membrane damage and can induce apoptosis and ferroptosis. (**F**) Peroxisomes generate ROS mainly through fatty acid β-oxidation, producing H_2_O_2_ that can diffuse into the cytosol when antioxidant capacity is exceeded. Under such conditions, peroxisomal ROS can amplify mitochondrial oxidative stress and promote apoptosis, induce autophagy through redox-sensitive signaling pathways, and enhance lipid peroxidation contributing to ferroptosis. ETC = electron transport chain; Fe^2+^ = ferrous; H_2_O_2_ = hydrogen peroxide; •OH = hydroxyl radicals; LOX = lipoxygenase; NOX = NADPH oxidase; ROS = reactive oxygen species. This figure was created with the assistance of BioRender (https://biorender.com); original files available on request.

Pharmacological efforts to modulate these ROS–RCD axes are already underway: ferroptosis inducers (such as erastin derivatives and GPX4 inhibitors) are being explored for drug-resistant cancers [329,330], whereas RIPK1-targeting necroptosis inhibitors (e.g., necrostatins, GSK2982772) and NLRP3 inflammasome blockers (such as MCC950) have entered preclinical and early clinical testing for inflammatory and degenerative diseases [331,332,333,334,335]. While these approaches highlight translational promise, their progress also illustrates major challenges, including context dependency of ROS signaling, pleiotropic drug effects, and the need for precise patient stratification. Beyond these exemplars, several modality-focused strategies are progressing clinically or preclinically across the ROS–RCD spectrum. For apoptosis, BCL-2 inhibitors such as venetoclax have reshaped standards of care in acute myeloid leukemia (AML) and continue to expand through combination regimens [336,337,338]. Notably, mitochondrial ROS production following BCL-2 inhibition has been implicated as a feed-forward amplifier of apoptosis. Moreover, the cellular redox state can modulate the efficacy of BCL-2 inhibitors: cells under high oxidative stress are more vulnerable to BCL-2 blockade, whereas robust antioxidant defenses can confer resistance [339,340]. This implies that combining Bcl-2 inhibitors with therapies that enhance oxidative stress, or weaken antioxidant systems, may improve their clinical efficacy. For autophagy-dependent cell death, lysosomotropic autophagy inhibitors (chloroquine/hydroxychloroquine) are under active clinical evaluation in oncology, with mixed but instructive results that underscore context-dependency and off-target liabilities. Importantly, their efficacy may partly derive from ROS accumulation upon autophagy blockade, because impaired clearance of damaged mitochondria fosters oxidative stress and amplifies cell death signals. This mechanism is supported by studies showing that autophagy inhibition increases sensitivity to oxidative stress in cancer models [341,342,343,344]. NETosis can be pharmacologically tempered in preclinical models by protein-arginine deiminase type-4 (PAD4) inhibition (e.g., GSK484), illustrating feasibility to reduce NET-driven inflammation. Mechanistically, PAD4 inhibition reduces citrullination of histones and disrupts PAD4–neutrophil cytosol factor (NCF) interactions, thereby attenuating NADPH oxidase-dependent ROS generation required for chromatin decondensation during NET formation [345]. However, alternative ROS-independent NETosis pathways also exist, which may limit complete suppression in vivo [239]. Methuosis-targeted strategies, such as pharmacological inhibition of PIKfyve (e.g., apilimod and related ligands), perturb lysosomal homeostasis and provoke vacuolation that can be modulated by ROS. Indeed, PIKfyve inhibition increases ROS production and oxidative stress, particularly affecting cathepsin susceptibility to oxidative modifications [346]. In addition, ROS have been shown to influence lysosomal dynamics under PIKfyve blockade by preventing lysosome coalescence and favoring fragmentation [347]. For lysosome-dependent cell death LDCD, targeting cathepsin activity or lysosomal integrity remains a promising but still nascent avenue. The interplay with ROS is supported by evidence that oxidative stress can compromise lysosomal membrane integrity, promoting leakage, cathepsin release, and initiation of downstream death pathways. For instance, cathepsin B has been implicated in diverse forms of programmed cell death and is considered a potential therapeutic target in disease settings [348]. Moreover, cancer-oriented strategies targeting lysosomal stability (e.g., lysosome membrane permeabilizers) are emerging as anticancer modalities, although their precise redox mechanisms remain under study [349]. These examples illustrate how therapeutic strategies can either buffer or harness ROS at organelle-specific sites, emphasizing the need to contextualize redox modulation within the broader network of RCD pathways (Figure 8). Since ROS generation is spatially and temporally compartmentalized across organelles and micro-domains, effective interventions will therefore require precise, pathway-informed redox modulation [319,326,350].

Beyond individual pathways, redox homeostasis is increasingly recognized as a central regulator of RCD, as many cell types operate close to their oxidative limits. Elevated ROS levels not only act as triggers but also modulate execution thresholds, thereby influencing the balance between membrane preservation or rupture, the inflammatory phenotype, and the switch between alternative RCD programs. A systematic understanding of these redox-sensitive checkpoints is therefore crucial to unravel the molecular logic by which ROS shape cell fate decisions. Ultimately, defining how ROS govern RCD at the mechanistic level will be essential for a coherent framework of RCD biology [2,351,352,353,354]. Future research will need to bridge mechanistic insights with therapeutic translation, harnessing ROS as both a vulnerability and a therapeutic lever. Achieving this will not only refine our understanding of cell death control but also open new avenues for precise interventions across cancer, inflammation, and degenerative disease.

## Figures and Tables

**Table 1:** Context-dependent requirement of ROS in apoptotic signaling.

Pathway	Condition/Stimulus	ROS Requirement	Mechanism	Key References
**Extrinsic**	ROS-dependent regulation of CD95L transcription (via NF-κB, AP-1)	Required	ROS sustain AP-1 (ASK1–JNK, c-Jun) and promote NF-κB nuclear translocation, enabling CD95L expression and receptor clustering	[108,115,119,120,121,122,123]
**Extrinsic**	Mitochondrial ROS (Complex I/III) driving CD95L induction	Required	Complex I/III-derived ROS enhance CD95L transcription, linking mitochondrial dysfunction with death receptor signaling	[46,68]
**Intrinsic**	ROS-mediated modulation of BCL-2 family and mitochondria	Required	mtROS drive cardiolipin oxidation, tBid docking, Bax/Bak activation, and support p53-dependent PUMA/NOXA induction → MOMP	[133,134,135,136,148]
**Intrinsic**	BH3 mimetics (venetoclax/navitoclax)	Optional	Direct antagonism of BCL-2 triggers MOMP; ROS act only as amplifiers	[149,150,151,152]
**Intrinsic**	DNA damage (p53-competent)	Optional	p53 induces PUMA/NOXA; apoptosis proceeds under ROS scavenging, with ROS accelerating responses	[136,148]
**Extrinsic**	Strong ligand stimulation (efficient DISC formation)	Optional	Suprathreshold receptor signaling drives caspase activation; ROS accelerate but are not essential	[39,153]
**Execution**	Excess ROS (supraphysiological)	Inhibitory	Oxidation of catalytic cysteines in caspases blocks execution; cells may deviate to necrosis/necroptosis	[143,144,154,155]

**Table 2:** Context-dependent requirement of ROS in necroptotic signaling.

Regulatory Level	Condition/Stimulus	ROS Requirement	Mechanism	Key References
**RIPK1 activation**	Mitochondrial ROS oxidize RIPK1 (Cys → pS161 autophosphorylation)	Required	mtROS drive RIPK1 activation and RIPK1–RIPK3 necrosome assembly; positive feedback loop	[165]
**Metabolic reprogramming**	RIPK3 phosphorylates pyruvate dehydrogenase → ↑ mitochondrial respiration/ROS	Required	RIPK3–MLKL axis enhances aerobic metabolism, boosting ROS to amplify necroptosis	[166,167]
**Checkpoint regulation**	Redox-dependent inhibition of caspase-8	Required	Caspase-8 oxidation disables apoptotic brake, permitting RIPK1–RIPK3 necrosome formation	[168,169,170]
**Transcriptional control**	NF-κB-dependent TNF, cIAPs, c-FLIP expression	Optional	Moderate ROS support NF-κB activity (pro-survival); excessive ROS block NF-κB DNA binding, impair IAPs, tilting balance to necroptosis	[117,122,147,173,174,175,176]
**Pharmacological modulation**	Trx-1 inhibition by dimethyl fumarate	Optional	Loss of redox buffering promotes Ripoptosome assembly; mixed apoptosis/necroptosis phenotype	[122,177,178]
**Execution phase**	Excess ROS beyond physiological range	Inhibitory/Shift	Overoxidation of NF-κB or metabolic collapse suppresses survival and apoptotic checkpoints, biasing outcome toward necroptosis	[122,175,176,179,180]

**Table 3:** Context-dependent requirement of ROS in ferroptotic signaling.

Regulatory Level	Condition/Stimulus	ROS Requirement	Mechanism	Key References
**Initiation**	Labile iron pool (Fe^2+^) + H_2_O_2_ → Fenton chemistry	Required	Fe^2+^/H_2_O_2_ generate •OH radicals that indiscriminately oxidize lipids, initiating ferroptosis	[41,185,186,187,188]
**Enzymatic** **amplification**	15-LOX–PEBP1 complex drives phosphatidylethanolamine peroxidation	Required	Enzymatic lipid peroxidation amplifies lipid ROS beyond detoxification capacity	[182,184]
**Transcriptional control**	Oncogenic/stress pathways p53 represses SLC7A11	Optional	ROS-induced stress and p53 signaling determine sensitivity to ferroptosis by regulating cystine uptake	[192,193]
**Execution phase**	Genetic or pharmacologic loss of GPX4	Required	Loss of GPX4 peroxidase activity causes lethal accumulation of lipid peroxides	[84,181,183,194,195,196]
**Context-specific modulation**	NF-κB inhibition (e.g., in CTCL)	Shift to ferroptosis	Disturbed iron/ROS homeostasis under NF-κB loss leads to ROS- and iron-dependent cell death	[53,189]

**Table 4:** Context-dependent requirement of ROS in pyroptotic signaling.

Regulatory Level	Condition/Stimulus	ROS Requirement	Mechanism	Key References
**Inflammasome activation**	Mitochondrial ROS promote NLRP3 assembly	Required	mtROS and mtDNA release amplify NLRP3 inflammasome formation and caspase-1 activation	[15,88,89]
**Inflammasome activation**	NOX-derived ROS drive priming and activation in phagocytes	Required/Context-dependent	Oxidative burst from NOX couples microbial sensing to inflammasome activation	[15,89,203]
**Inflammasome activation**	TXNIP released upon TRX oxidation binds NLRP3	Required	TXNIP acts as a redox-sensitive switch linking oxidative imbalance to caspase-1 activation and pyroptosis	[201,204,205,206]
**Additional ROS sources**	Peroxisomal H_2_O_2_	Optional	Peroxisomes contribute H_2_O_2_ as a cofactor in inflammasome signaling	[15]

**Table 5:** Context-dependent requirement of ROS in lysosome-dependent cell death (LDCD).

Regulatory Level	Condition/Stimulus	ROS Requirement	Mechanistic Handle (One-Liner)	Key References
**Lysosomal ROS generation**	Intra-lysosomal Fenton chemistry (H_2_O_2_ + Fe^2+^ → •OH)	Required	Hydroxyl radicals attack lysosomal membranes, destabilizing integrity and triggering LMP	[228,229]
**Protective modulation**	Lysosomotropic iron chelators	Optional	Chelation prevents ROS-driven lipid peroxidation and cathepsin release	[230]
**Apoptotic outcome (limited LMP)**	Partial cathepsin release → BID cleavage → MOMP → caspase activation	Shift to apoptosis	Moderate ROS promote controlled LMP, linking lysosomes to mitochondrial apoptosis	[21,223,231]
**Necrotic outcome (extensive LMP)**	Massive cathepsin/hydrolase leakage → direct proteolysis	Required	Excessive ROS cause catastrophic lysosomal rupture and caspase-independent necrotic cell death	[21,223]
**Repair/clearance balance**	ESCRT machinery and lysophagy counteract LMP	Shift	When repair or lysophagy fails, ROS-induced LMP tilts balance toward LDCD	[224,225,226,227]

**Table 6:** Context-dependent requirement of ROS in NETosis.

Regulatory Level	Condition/Stimulus	ROS Requirement	Mechanism	Key References
**Canonical** **(Suicidal NETosis)**	NOX2-derived oxidative burst	Required	NOX2-dependent ROS drive NE and MPO translocation to the nucleus, promoting chromatin decondensation and NET release; absent in chronic granulomatous disease	[90,235,236,237]
**Mitochondrial ROS (vital NETosis)**	Mitochondrial ROS and Ca^2+^ influx	Required/Context-dependent	Mitochondrial ROS trigger rapid NET release while preserving neutrophil viability (vital NETosis)	[234]
**ROS-independent** **NETosis**	Physiological agonists (Ca^2+^ ionophores, monosodium urate crystals)	Absent/Alternative pathway	Certain stimuli induce NET release without detectable ROS burst, indicating stimulus-specific, ROS-independent NETosis	[238,239,240]

**Table 7:** Context-dependent requirement of ROS in autophagy.

Regulatory Level	Condition/Stimulus	ROS Requirement	Mechanism	Key References
**Initiation (nutrient stress)**	Starvation-induced oxidation of Atg4	Required	ROS oxidize Atg4 to enable LC3 processing and autophagosome formation	[259,260]
**Energy-sensing pathway**	AMPK/ULK1 activation under oxidative stress	Required/Context-dependent	ROS activate AMPK and ULK1, promoting autophagy initiation and metabolic adaptation	[253,254]
**Regulation via Bcl-2/Beclin-1 and KEAP1 degradation**	Moderate oxidative stress	Required/Context-dependent	ROS disrupt Bcl-2–Beclin-1 complexes and trigger KEAP1 degradation, enhancing Nrf2-dependent antioxidant responses	[253,254]
**Negative feedback control (mitophagy, pexophagy)**	Selective removal of ROS-producing organelles	Optional/Feedback	Autophagy reduces ROS burden via degradation of damaged mitochondria or peroxisomes	[253]
**Autophagy-dependent cell death (ADCD/AMCD)**	Sustained or excessive ROS accumulation	Required	Persistent ROS drive destructive, non-protective autophagy contributing directly to cell death	[255,256,257,258]
**mTOR inhibition (rapamycin-induced autophagy)**	Pharmacological mTOR blockade	ROS-independent	Rapamycin triggers autophagy even under antioxidant conditions, indicating ROS-independent induction	[261,262]

**Table 8:** Comparative overview of regulated cell death (RCD) modalities with respect to plasma membrane integrity and inflammatory potential.

RCD Modality	Plasma Membrane Integrity	Inflammatory Potential
**Apoptosis**	Preserved	Non-inflammatory
**Anoikis**	Preserved	Non-inflammatory
**Necroptosis**	Ruptured	Inflammatory
**Ferroptosis**	Ruptured	Inflammatory
**Pyroptosis**	Ruptured	Inflammatory
**Paraptosis**	Ruptured	Inflammatory
**Parthanatos**	Ruptured	Inflammatory
**LDCD**	Ruptured	Inflammatory
**Oxeiptosis**	Preserved	Non-inflammatory
**suicidal NETosis**	Ruptured	Inflammatory
**Vital NETosis**	Preserved	Inflammatory (controlled release of DAMPs)
**Autophagy**	Preserved	Non-inflammatory
**MPT-driven necrosis**	Ruptured	Inflammatory
**Entotic cell death**	Preserved	Non-inflammatory
**Mitotic catastrophe**	Variable (often progresses to apoptosis → preserved)	Non-inflammatory
**Mitotic death**	Preserved	Non-inflammatory
**Cuproptosis**	Ruptured	Inflammatory
**Alkaliptosis**	Ruptured	Inflammatory
**Methuosis**	Ruptured (late vacuole collapse)	Inflammatory
**Disulfidptosis**	Likely ruptured	Likely inflammatory

## Data Availability

No new data were created or analyzed in this study. Data sharing is not applicable to this article.
